# Supplementary material for: Analyses of a chromosome-scale genome assembly reveal the origin and evolution of cultivated chrysanthemum
Source: Nat Commun. 2023 Apr 11;14:2021. doi: 10.1038/s41467-023-37730-3 (PMC10085997; doi:10.1038/s41467-023-37730-3)
Supplement: Supplementary file 1 — Supplementary Information [file 41467_2023_37730_MOESM1_ESM.pdf]

Analyses of a chromosome-scale genome assembly reveal the origin and evolution of cultivated chrysanthemum

Song *et al*.

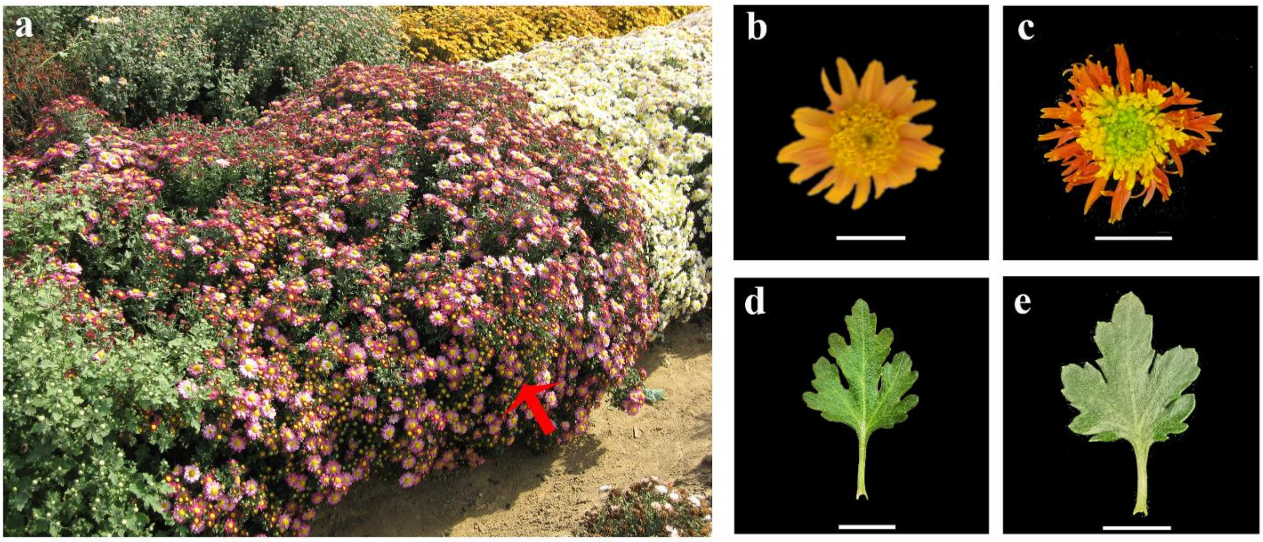

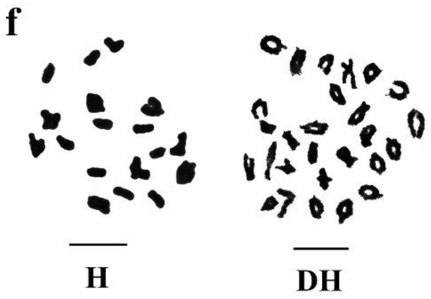

Taxa	Mean chromosome pairing configuration per PMC						
	No. of PMCs observed	Univalent (Ⅰ)	Bivalent (Ⅱ)			Trivalent (Ⅲ)	Tetravalent (Ⅳ)
			Bar	Ring	Total		
**Haploid** (n=3x=27)	15	9.13	6.33	2.6	8.93	0.00	0.00
**Doubled haploid** (2n=6x=54)	20	0.6	8.45	17.35	25.80	0.00	0.45

Supplementary Figure 1. Morphological and cytological characteristics of *C. morifolium* cv. 'Zhongshanzigui' and its haploid and doubled haploid derivatives. a The phenotype of pot chrysanthemum variety 'Zhongshanzigui' (2n=6x=54) at flowering stage. The flower (**b**) and leaf (**d**) morphology of 'Zhongshanzigui' haploid (H) plant, which was obtained from *in vitro* ovules culture and used for genome sequencing. The flower (**c**) and leaf (**e**) morphology of doubled haploid (DH) plant that was induced by colchicine treatment. Bar (**b-e**) = 1 cm. The details on materials creation could be found in Wang *et al*.[1] **f** Metaphase I chromosome pairing configurations in PMCs (pollen mother cells) of the haploid (9II + 9I) and doubled haploid (27II) plants. Bar = 10 μm.

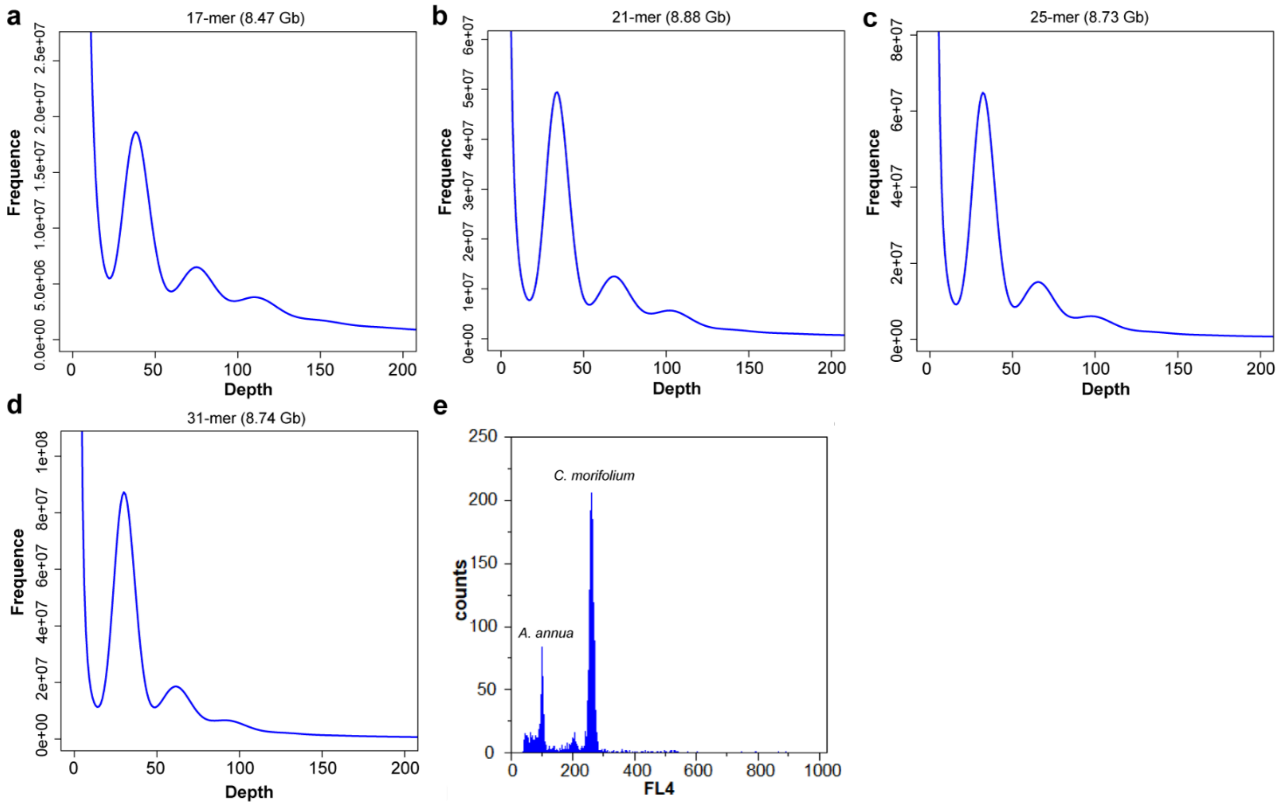

Supplementary Figure 2. Evaluation of *C. morifolium* genome size by *K*-mer and cell flow cytometry analyses. a-d Distribution of 17-mer, 21-mer, 25-mer, 31-mer in Illumina sequence data of 'Zhongshanzigui' haploid. The x-axis and y-axis separately indicate the *K*-mer depth and frequency, respectively. The genome size shown in the brackets for each kmer was respectively calculated using the frequency peak at 39, 34, 33, 31 as coverage depth according to the following formula: genome size = total *K*-mer count / coverage depth. The other two peaks at ~75× and ~110× indicates a potential hexaploid genome of this species. **e** Relative 1C DNA content measured by cell flow cytometry, X axis shows the relative DNA content and the Y axis shows the strength of fluorescence signal calculated as the number of events. We used the *Artemisia annua* genome (1.74 Gb) as a reference. The genome size of *C. morifolium* was estimated to be 9.02 Gb.

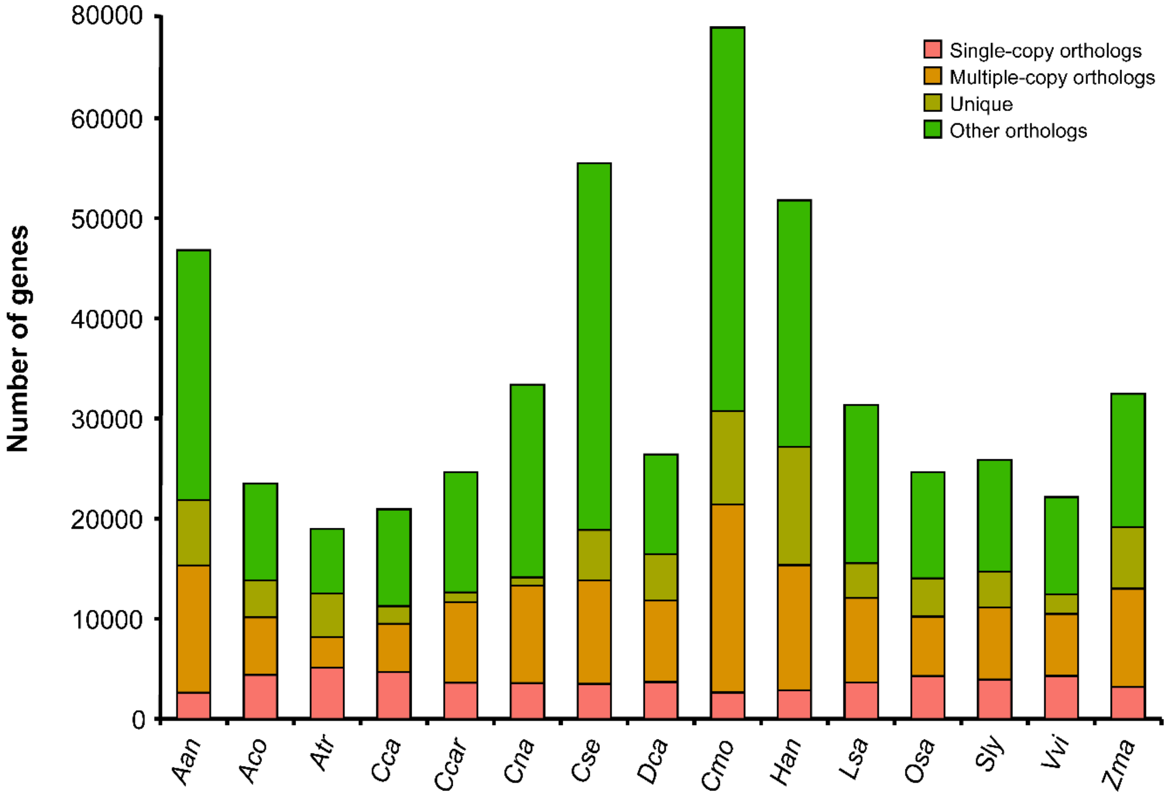

Supplementary Figure 3. Comparisons of ortholog protein families among the 15 plant species used for analysis. The scientific name of each species is list in Supplementary Data 3. Given each species must have at least one gene within a gene family, if there is only one member for a certain species, it is regarded as a single-copy ortholog, else it is regarded as a multiple-copy ortholog. Unique ortholog indicates species-specific gene family. Besides above, given the number of species having at least one gene family member smaller than the total species number, if there is at least one member within this gene family for a certain species, it is regarded as other ortholog.

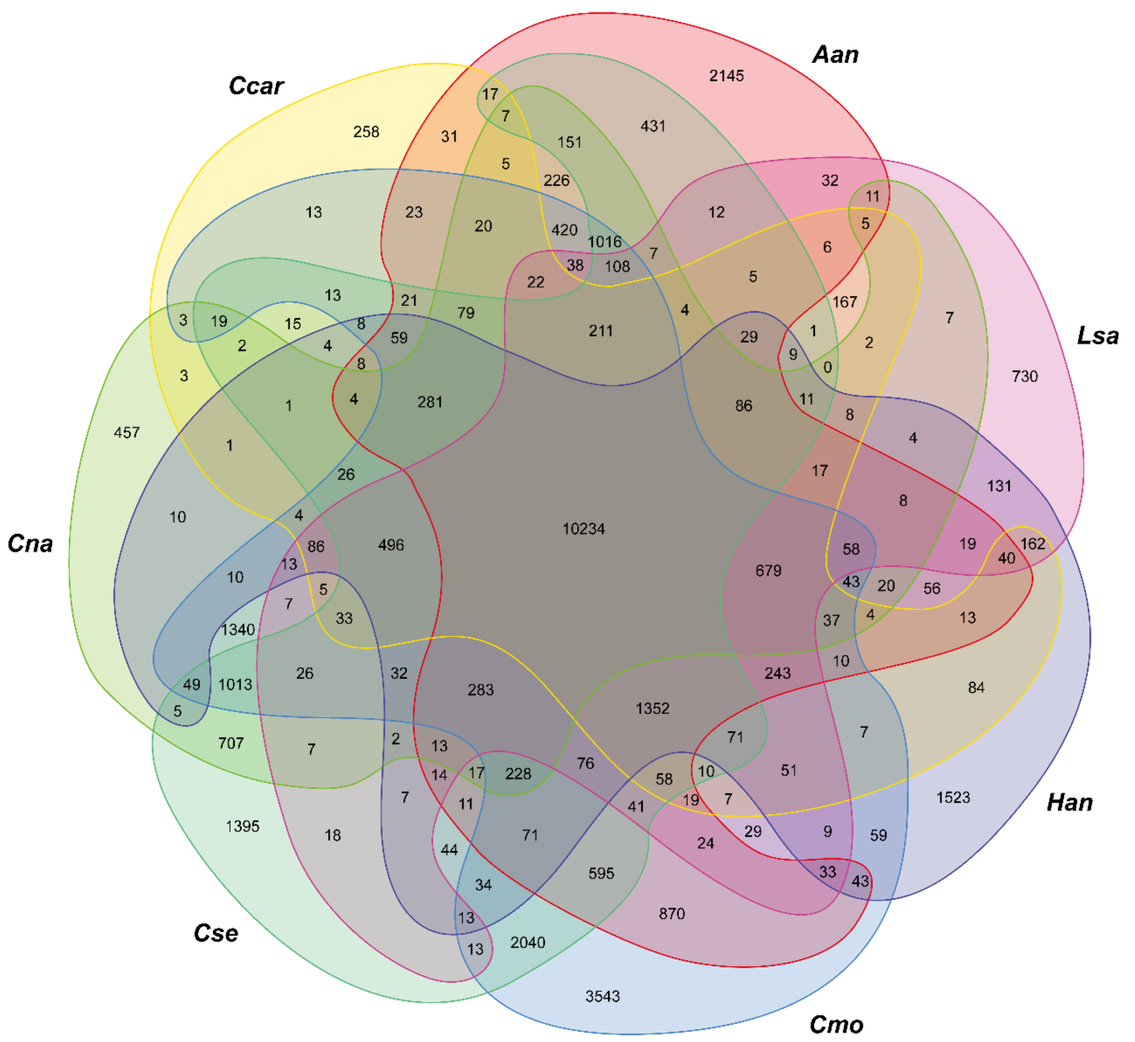

Supplementary Figure 4. Venn diagram represents the shared and unique gene families among seven Asteraceae plants. The scientific name of each species is list in Supplementary Data 3.

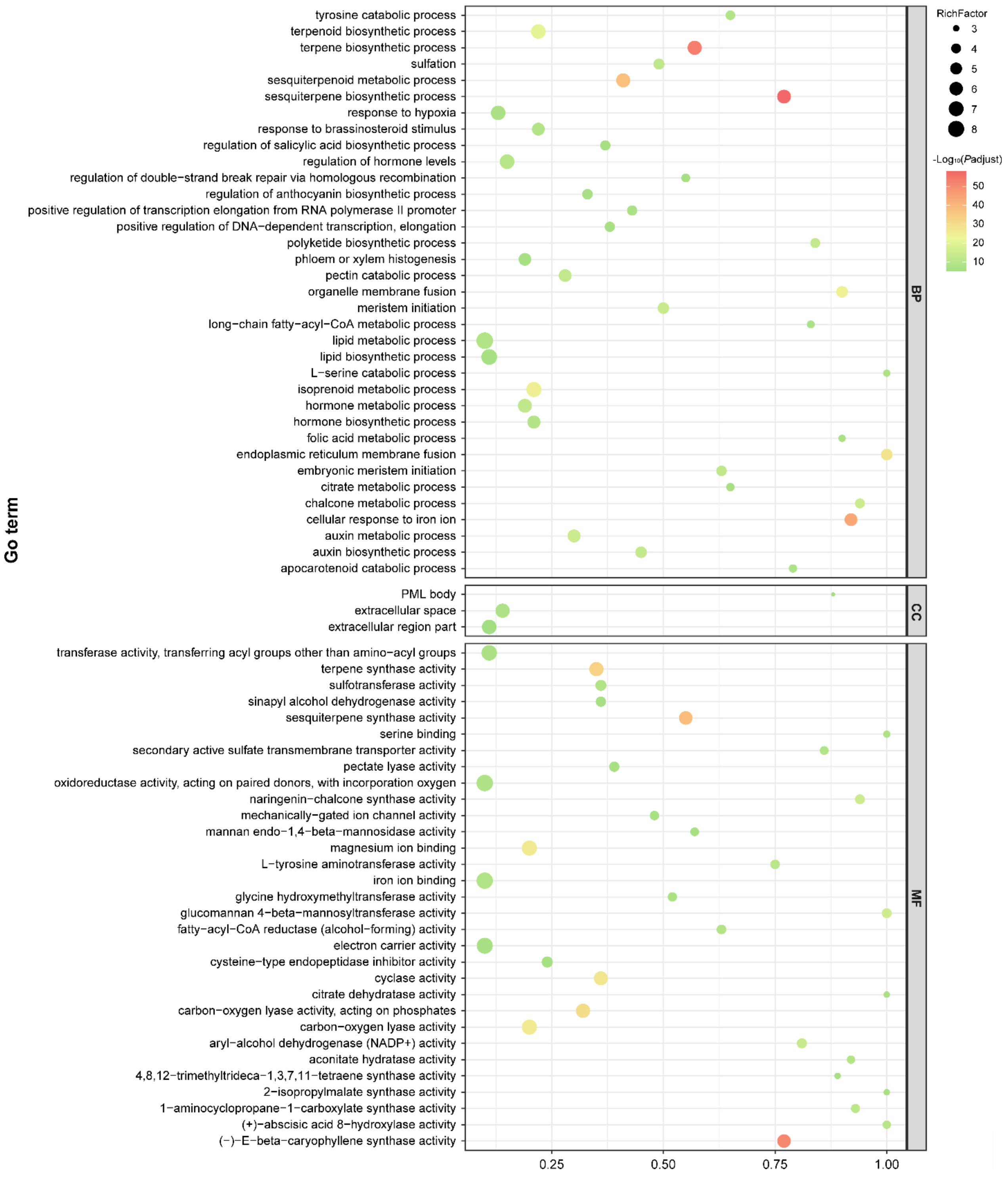

Supplementary Figure 5. GO functional enrichment analysis for the chrysanthemum expanded genes. *P* values are adjusted using a two-sided hypergeometric test followed by false discovery rate (FDR) correction for multiple testing. The enriched GO terms of biological process (BP), cellular component (CC) and molecular function (MF) with corrected *P* value < 1E-6 are presented. The colour of circles represents the statistical significance of enriched GO terms. The size of the circles indicates the number of genes in a GO term. Detailed information is shown in Supplementary Data 4 .

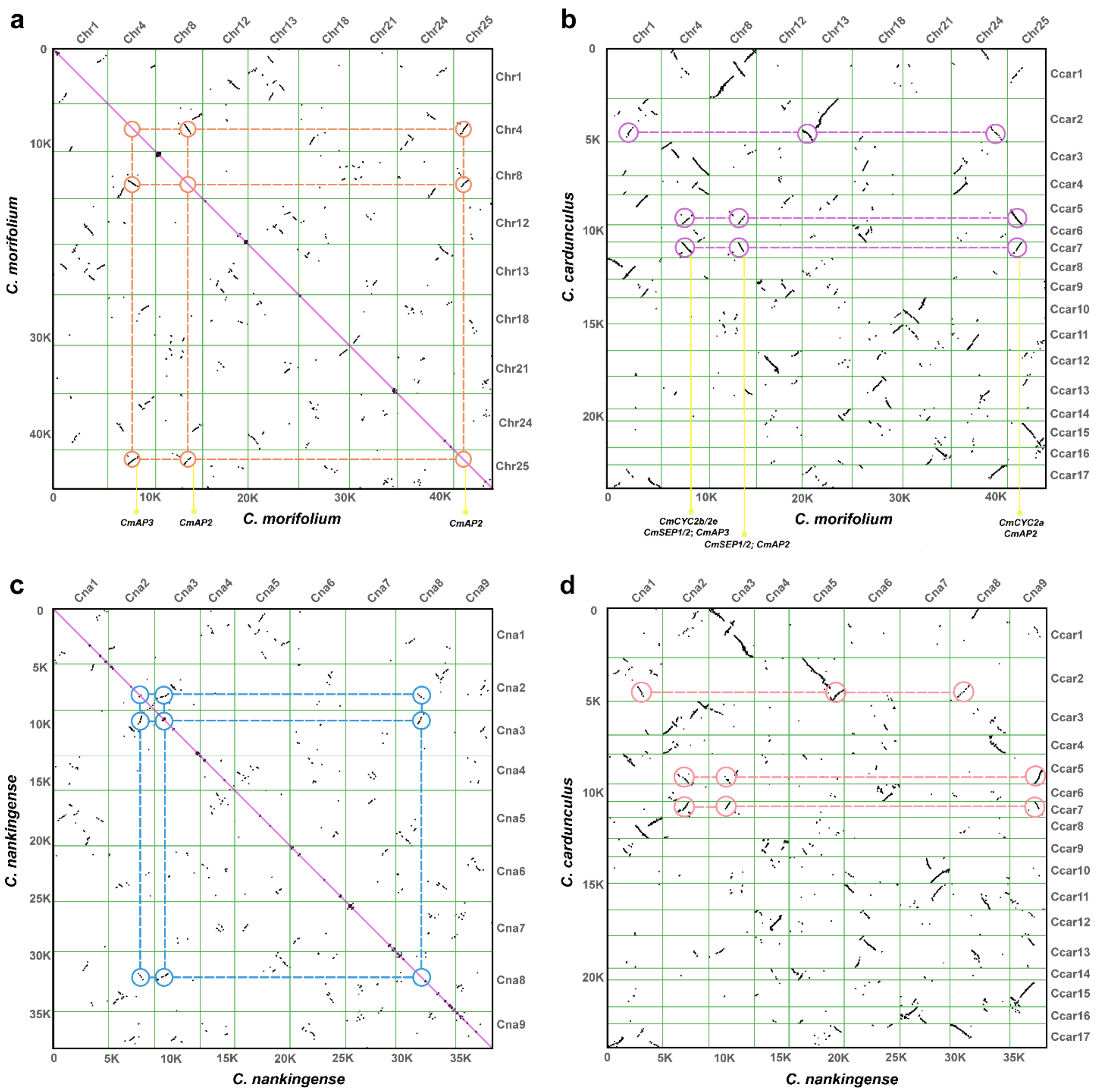

Supplementary Figure 6. Dot plots showing the syntenic relationships among *C. morifolium*, *C. carduncullus* and *C. nankingense.* **a** Intra-genomic comparison within *C. morifolium* monoploid genome based on 2,902 paralogous gene pairs. Here we selected the longest pseudochromosomes within each homoeologous group (Chr1, Chr4, Chr8, Chr12, Chr13, Chr18, Chr21, Chr24, and Chr25) to represent the ancestral genome of cultivated chrysanthemum in this analysis. Some syntenic blocks between pseudochromosomes are circled in orange, indicating the WGT-2 event. **b** Inter-genomic comparison between *C. morifolium* and *C. carduncullus* using 18,336 orthologous gene pairs. The 3 to 1 syntenic relationships are denoted in purple circles. The chromosomes of *C. carduncullus*, which experienced only the Asteraceae-shared WGT-1 event, are named Ccar1 through Ccar17. **c** Intra-genomic comparison within *C. nankingense* based on 2,494 paralogous gene pairs. Some syntenic blocks between pseudochromosomes are circled in blue, providing the evidence for the recent *Chrysanthemum* common WGT-2 event. **d** Inter-genomic comparison between *C. nankingense* and *C. carduncullus* using 16,808 orthologous gene pairs. Some 3 to 1 syntenic relationships are denoted in pink circles. The genes related to flower development in the syntenic regions are shown in **a** and **b**.

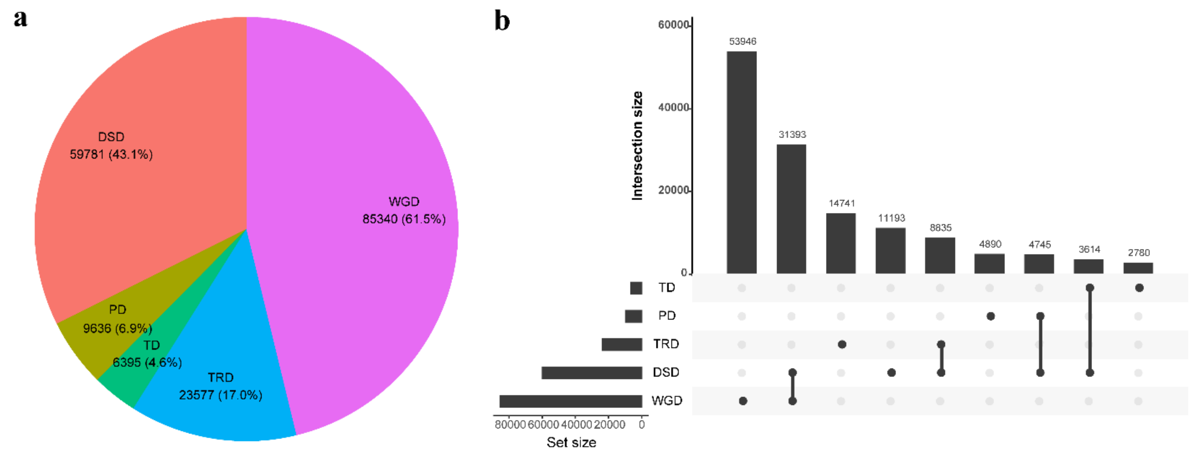

Supplementary Figure 7. The distribution of five modes of duplicate genes in *C. morifolium*. a The proportions of five modes of gene duplications. **b** Gene UpSet plot of duplicate genes. WGD, whole-genome duplication; TD, tandem duplication; PD, proximal duplication; TRD, transposed duplication; DSD, dispersed duplication. Source data are provided as Source Data file.

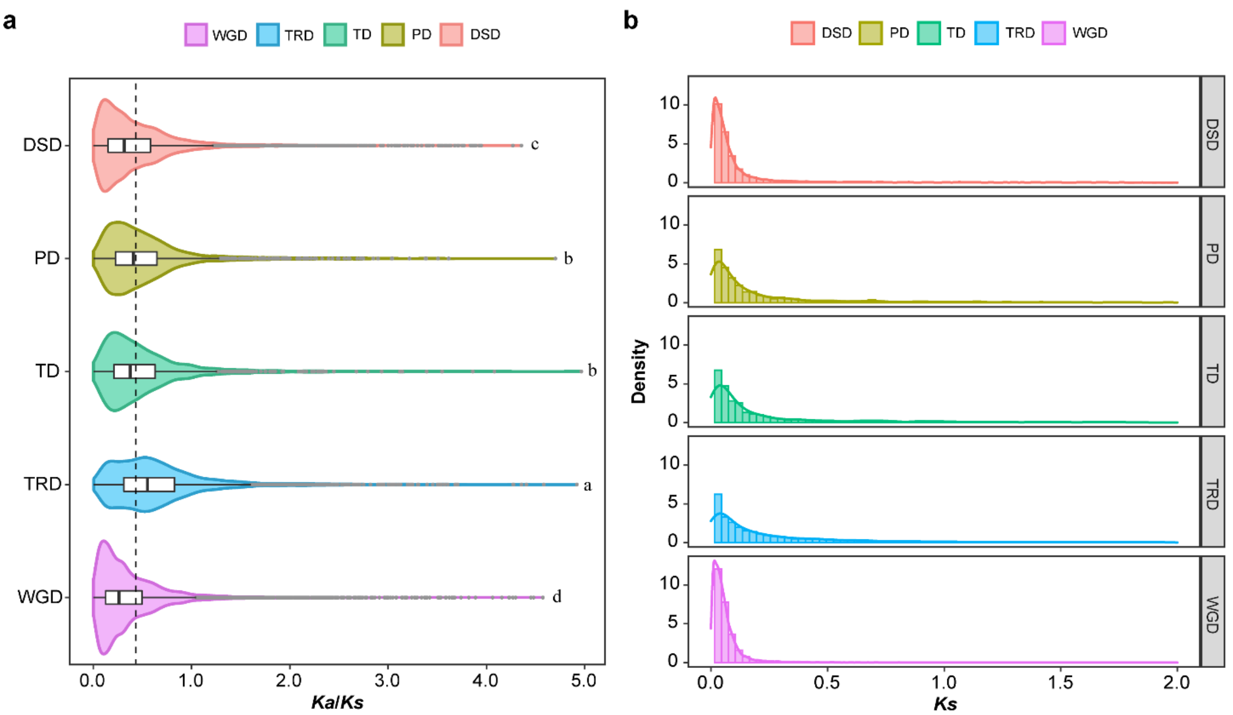

Supplementary Figure 8. Gene duplication and evolution. a The *Ka*/*Ks* ratio distributions of gene pairs derived from five modes of duplication. The central line for each box plot is the median value, the top and bottom edges correspond to the 25th and 75th percentiles, and the whiskers represent the 1.5 times the inter-quartile range (IQR) extending from the edges of the box. The grey points are outliers. The black dotted line indicates the average *Ka*/*Ks* value of the five modes of duplicated genes. A two-tailed Tukey's honestly significant difference (HSD) multiple comparison test was used to determine the significance (means with different lowercase letters are significantly different at $P < 0.01$). n = 20,050, 1,460, 1,855, 4,078, 13,161 for WGD, TD, PD, TRD, DSD, respectively. **b** *Ks* distribution of the five modes of gene duplications. WGD, whole genome duplication; TD, tandem duplication; PD, proximal duplication; TRD, transposed duplication; DSD, dispersed duplication.

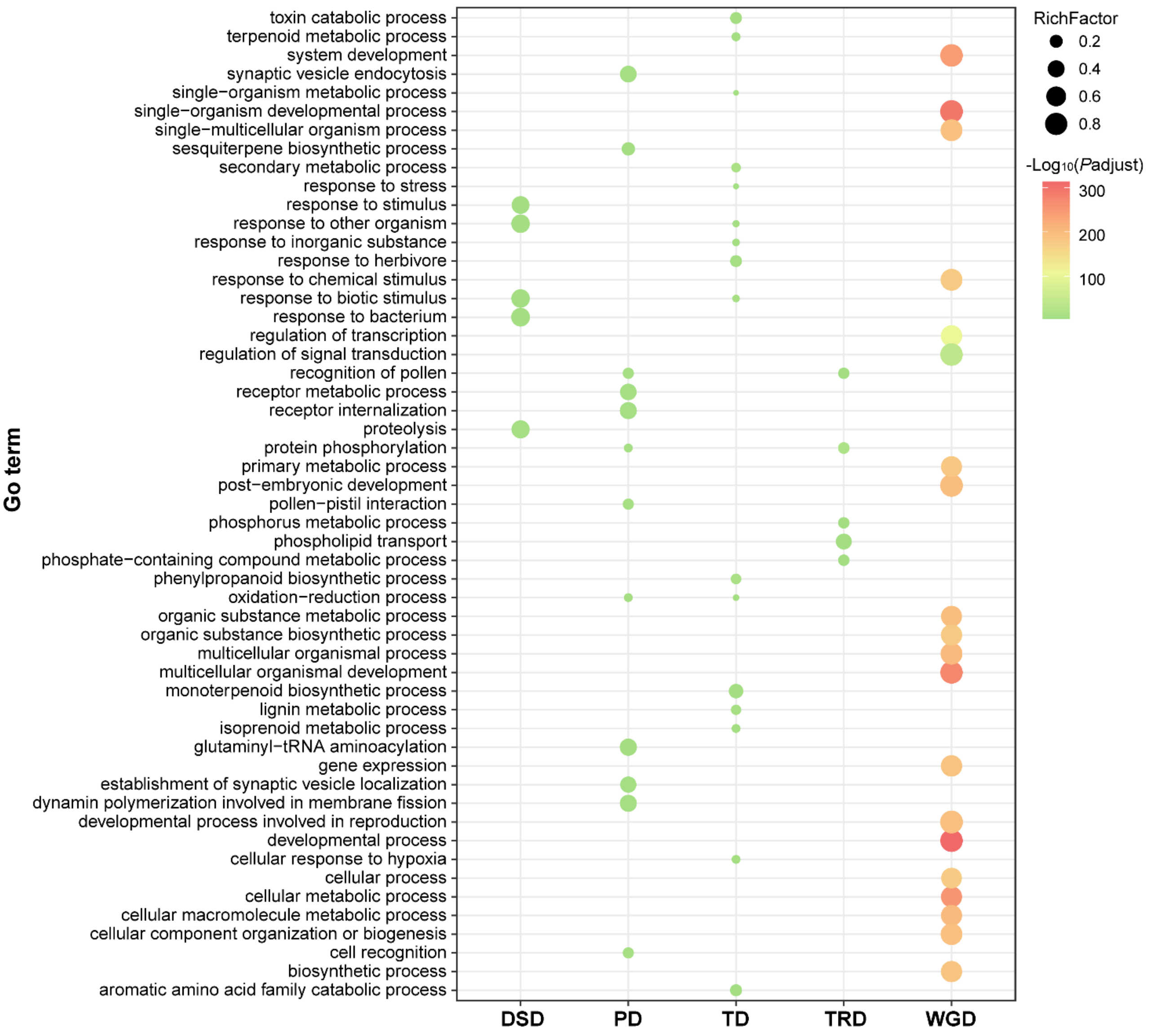

Supplementary Figure 9. GO functional enrichment analysis of the five modes of duplicate genes (DSD, PD, TD, TRD, and WGD). *P* values are adjusted using a two-sided hypergeometric test followed by false discovery rate (FDR) correction for multiple testing. The top 20 GO term (biological process) enrichment results with smallest corrected *P* value are presented. The colour of circles represents the statistical significance of enriched GO terms. The size of the circles indicates the proportion of enriched genes in a given GO term. For all annotated genes are provided as background information.

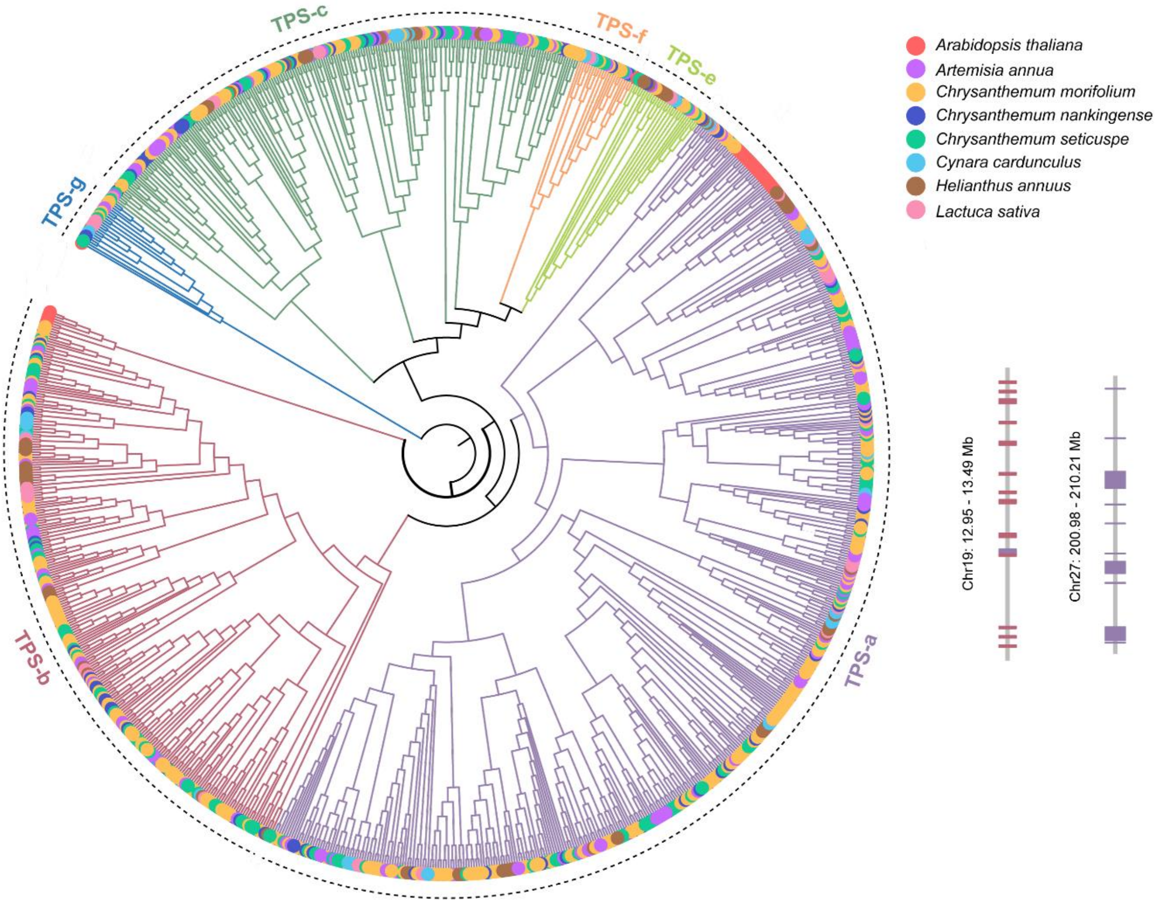

Supplementary Figure 10. Phylogeny of TPSs identified in *C. morifolium* and 7 other sequenced plant genomes showing the subfamilies from a-g. The TPS proteins of *C. morifolium* are marked by the orange point. Right panel shows details of the TPS-b (red brown-coded) and TPS-a (purple-coded) clusters on Chromosomes 19 and 27, respectively.

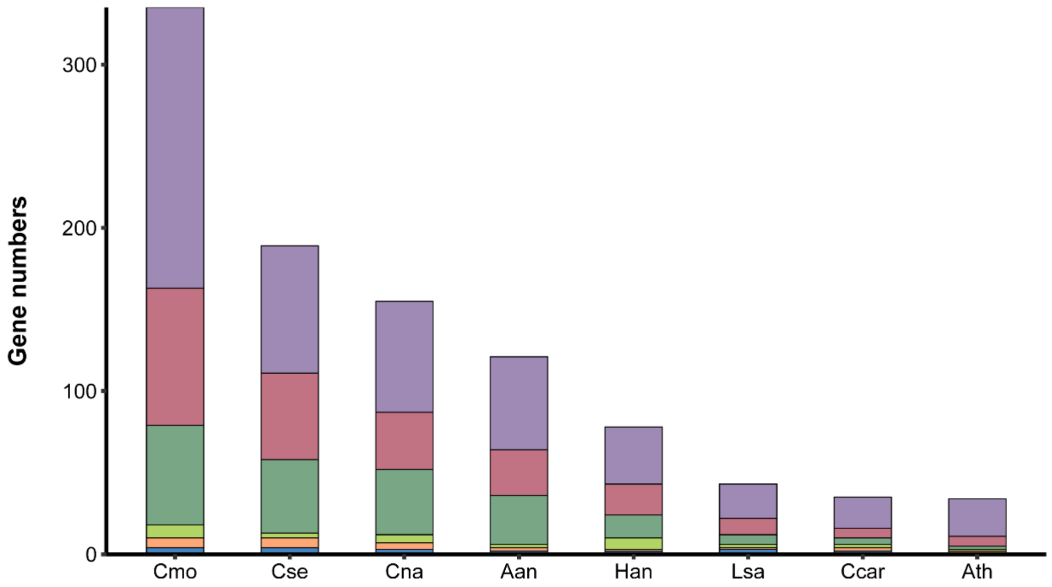

	Cmo	Cse	Cna	Aan	Han	Lsa	Ccar	Ath
Total	335	155	189	121	78	43	35	34
TPS−a	172	68	78	57	35	21	19	23
TPS−b	84	35	53	28	19	10	6	6
TPS−c	61	40	45	30	14	6	4	2
TPS−e	8	5	3	2	7	2	2	1
TPS−f	6	4	6	2	1	1	2	1
TPS−g	4	3	4	2	2	3	2	1

Supplementary Figure 11. Comparisons of TPS protein families among *C. morifolium* and 7 other sequenced plant genomes. The scientific name of each species is list in Supplementary Data 3.

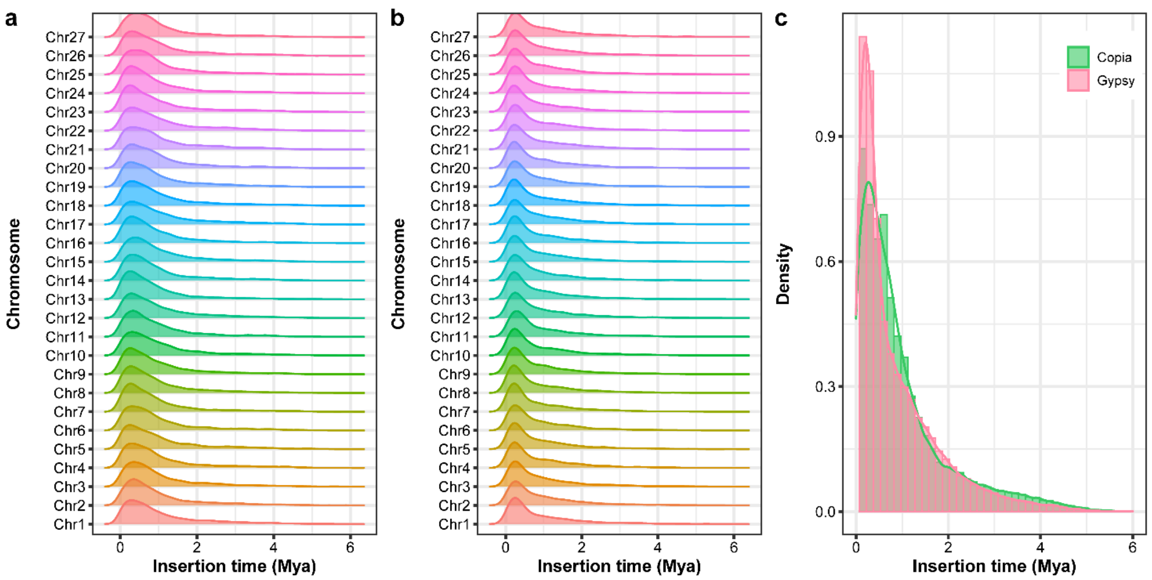

Supplementary Figure 12. Insertion times of LTR retrotransposons of *Copia* and *Gypsy* in *C. morifolium*. a Insertion time of the *Copia* family in the centromeric regions on each chromosome. **b** Insertion time of the *Gypsy* family in the centromeric regions on each chromosome. **c** Insertion time of *Copia* (green) and *Gypsy* (pink) families on family levels.

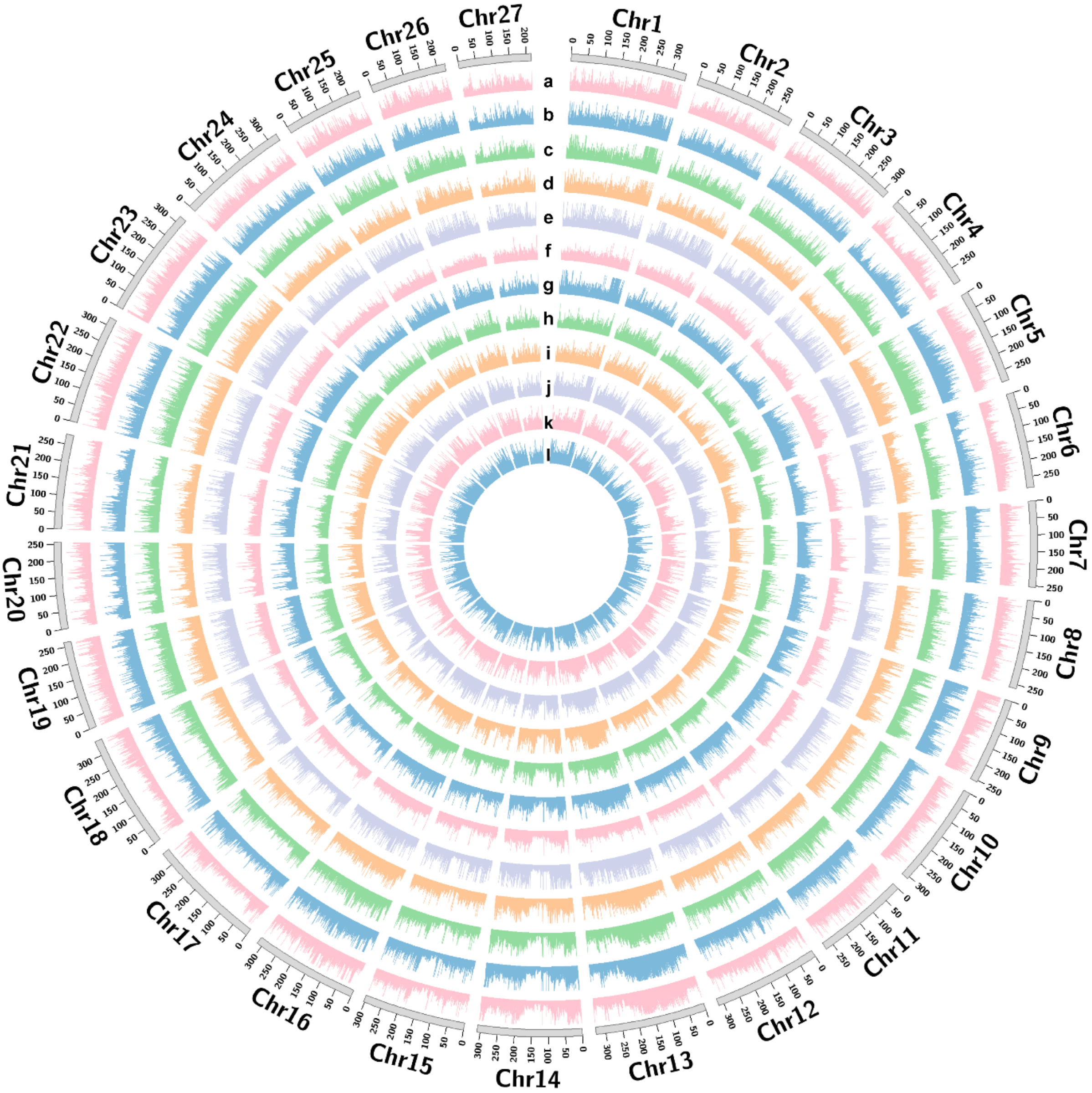

Supplementary Figure 13. Coverage depth of the mapped reads of 12 Chinese *Chrysanthemum* species with reference to the assembled *C. morifolium* genome. Chromosomes were binned into 100 kb non-overlapping sliding windows to display the average depth along assembled chromosomes. The tracks **a**~l indicate the coverage depth of *C. lavandulifolium*, *C. nankingense*, *C. indicum* (Henan), *C. indicum* (Hubei), *C. rhombifolium*, *C. indicum* (Nanjing), *C. indicum* (Shennongjia), *C. indicum* (Tianzhushan), *C. potentilloides*, *C. indicum* (Wuyishan), *C. dichrum*, *C. indicum* (Yuntaishan), respectively. Source data are provided as a Source Data file.

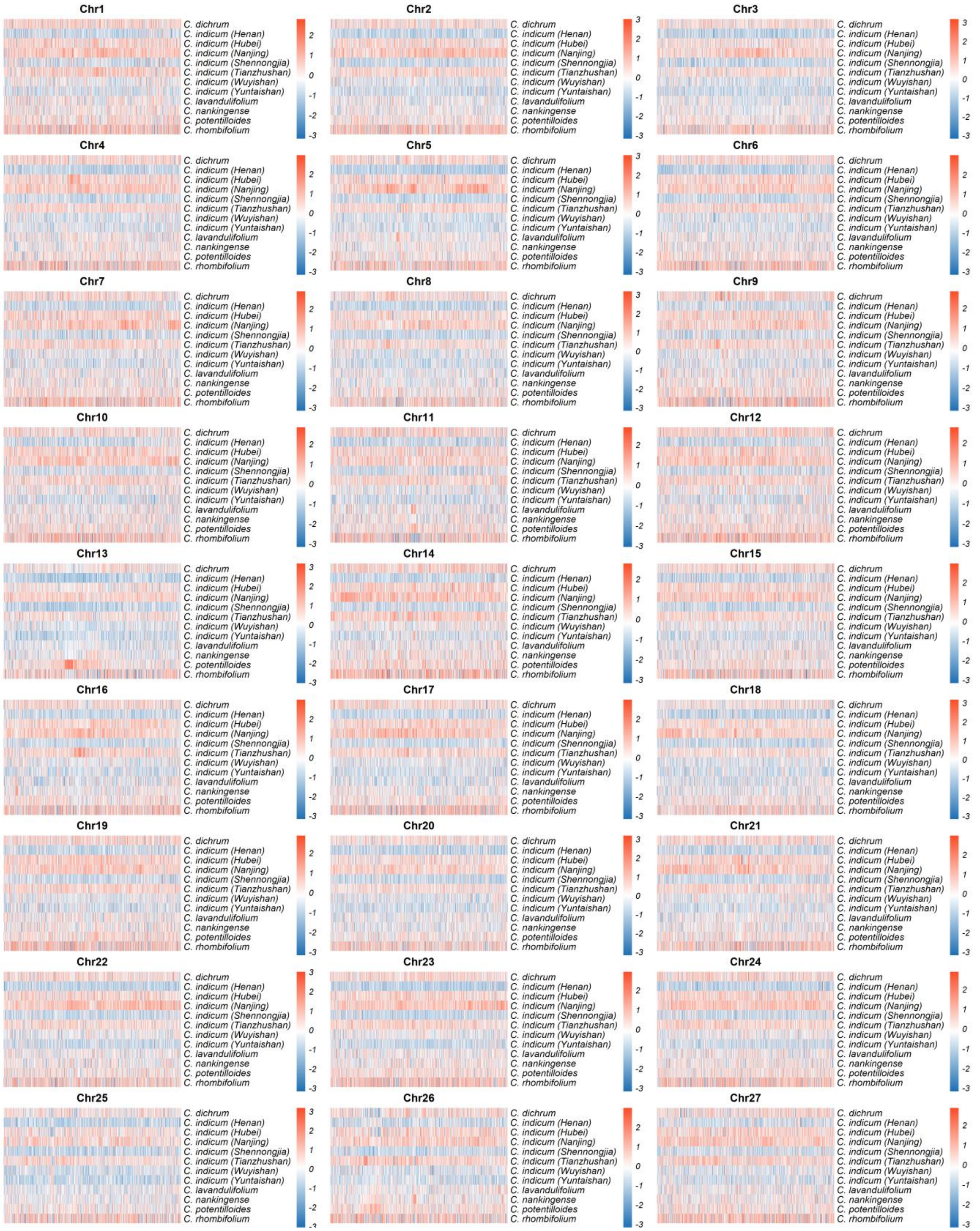

Supplementary Figure 14. Genomic similarity of 12 Chinese wild species of *Chrysanthemum* to the 'Zhongshanzigui' reference genome. Identical score (IS) values are calculated for SNPs within each 100 kb window across the genome. Scaled IS values in each window are shown from red to blue in colour, indicating high (red) to low (blue) genomic similarity of wild *Chrysanthemum* species to the 'Zhongshanzigui' reference genome.

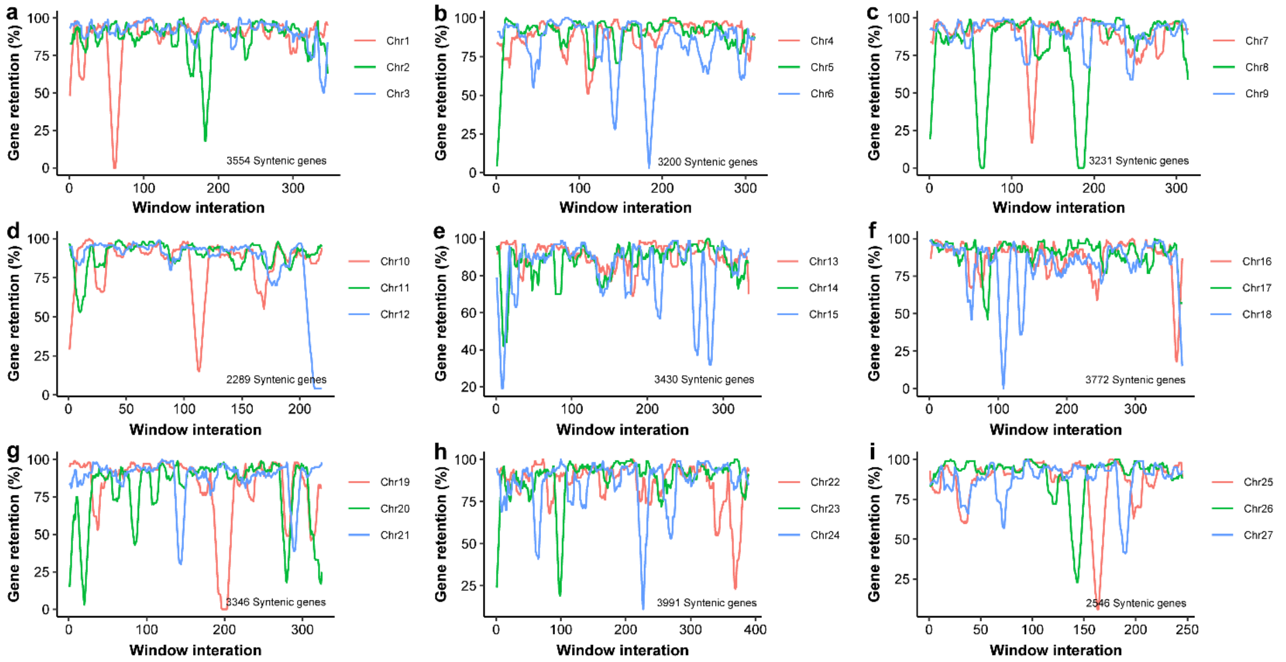

Supplementary Figure 15. Gene retention patterns among the nine homoeologous groups in *C. morifolium* using diploid *C. nankingense* genome as reference. a~i A sliding window approach with window size of 100 syntenic genes and step size of 10 syntenic genes was used to show the percentage of retained genes in different set of homoeologous groups of *C. morifolium* using diploid *C. nankingense* genome assembled in the present study as reference. In total, there were 29,359 (77.27%) genes in *C. nankingense* that were syntenic with at least one subgenome in *C. morifolium*, among which 22,337 (~76.08%) syntenic genes had three homoeologous copies retained in *C. morifolium*.

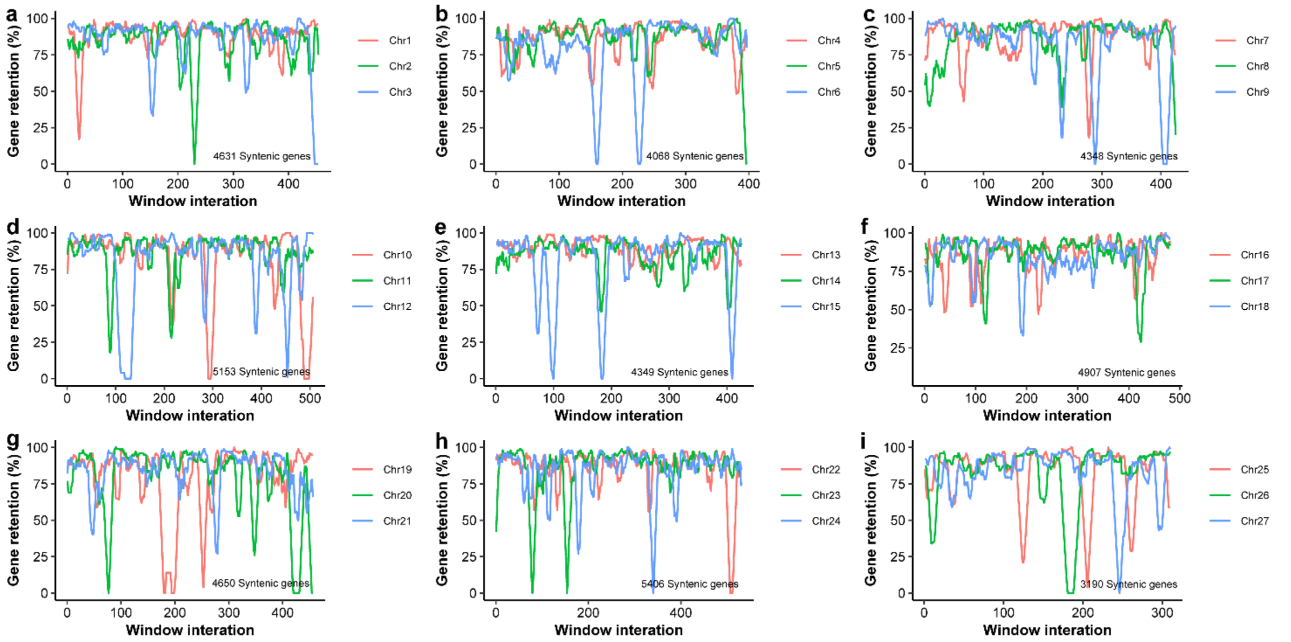

Supplementary Figure 16. Gene retention patterns among the nine homoeologous groups in *C. morifolium* using diploid *C. seticuspe* genome as reference. a~i A sliding window approach with window size of 100 syntenic genes and step size of 10 syntenic genes was used to show the percentage of retained genes in different set of homoeologous groups of *C. morifolium* using the released chromosome-level genome of *C. seticuspe* (Nakano *et al.*[2]) as reference. In total, there were 40,702 (54.81%) genes in *C.seticuspe* that were syntenic with at least one subgenome in *C. morifolium*, among which 25,900 (~63.63%) syntenic genes had three homoeologous copies retained in *C. morifolium*.

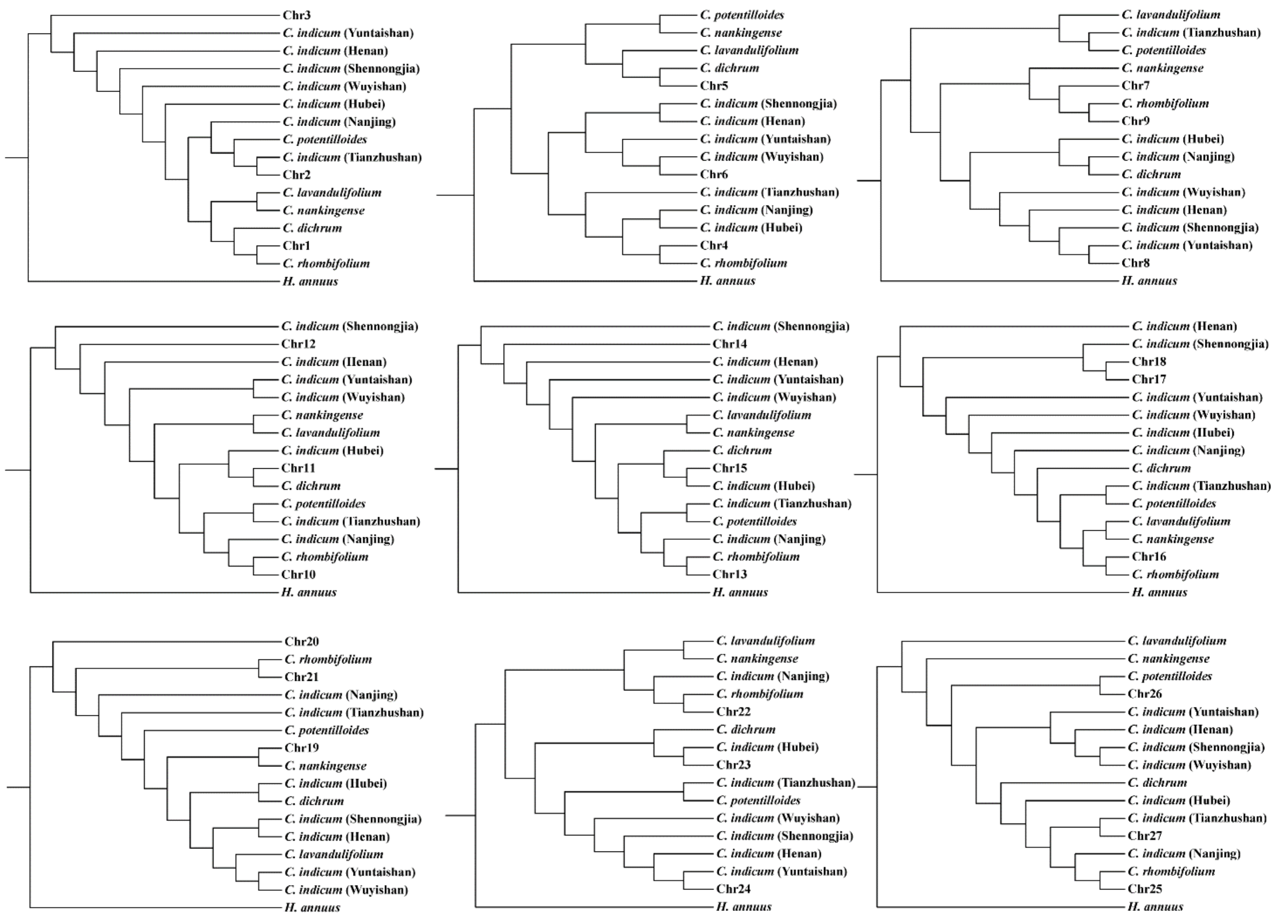

Supplementary Figure 17. Maximum-likelihood phylogenetic tree of *Chrysanthemum* for each of the nine homoeologous groups, using sunflower (*H. annuus*) as an outgroup.

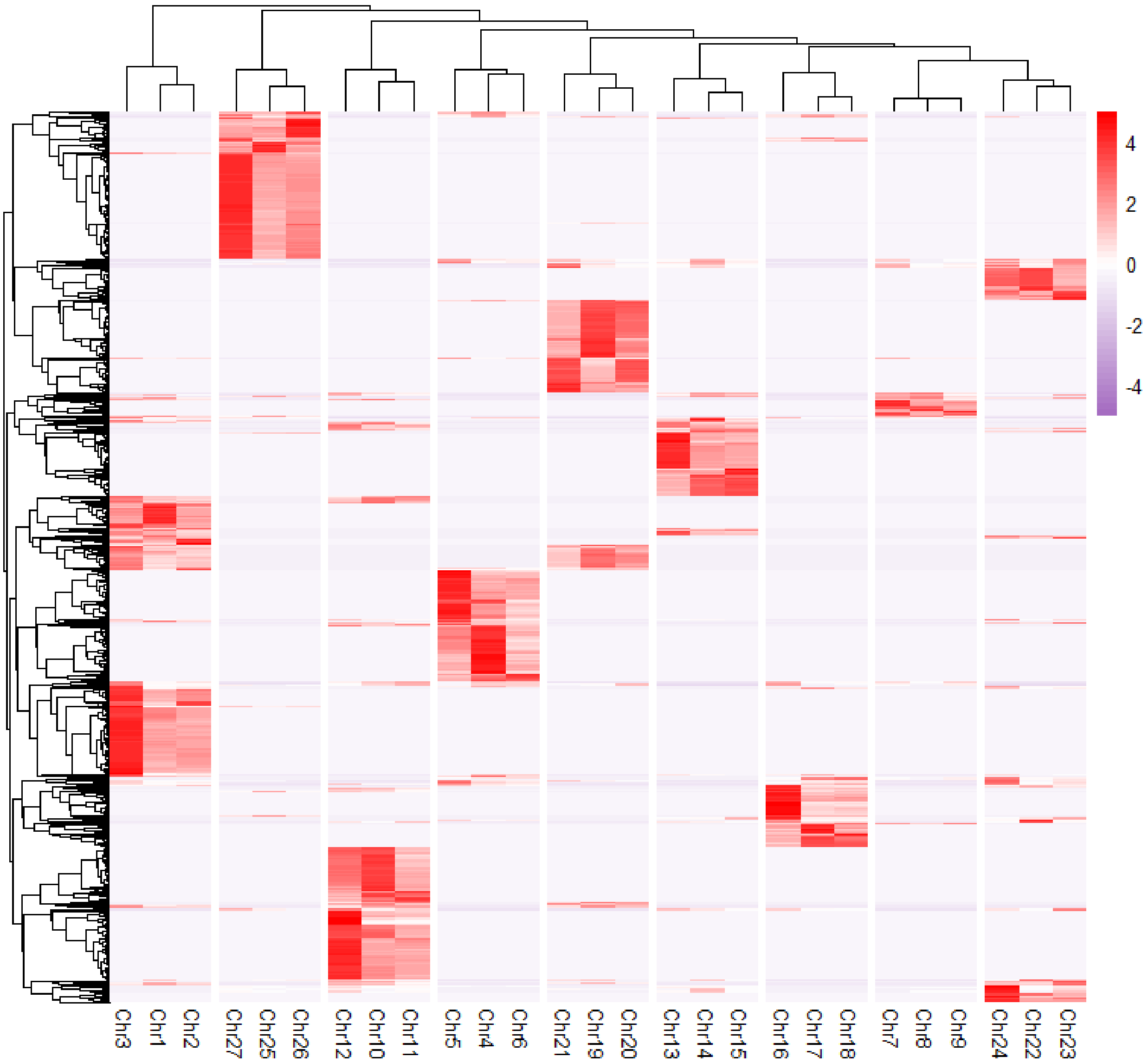

Supplementary Figure 18. Clustering of 13-mers counts enables the consistent partitioning of the *C. morifolium* genome into nine distinct homoeologous groups. We scanned the 27 pseudochromosomes for 13-bp sequences (13-mers) that (1) were found occurring at least 100 times across the whole *C. morifolium* genome, and (2) for each homoeologous group, were at least two-fold enriched in one member relative to either two pseudochromosomes. Thus, a total of 4,719 13-mers were identified.

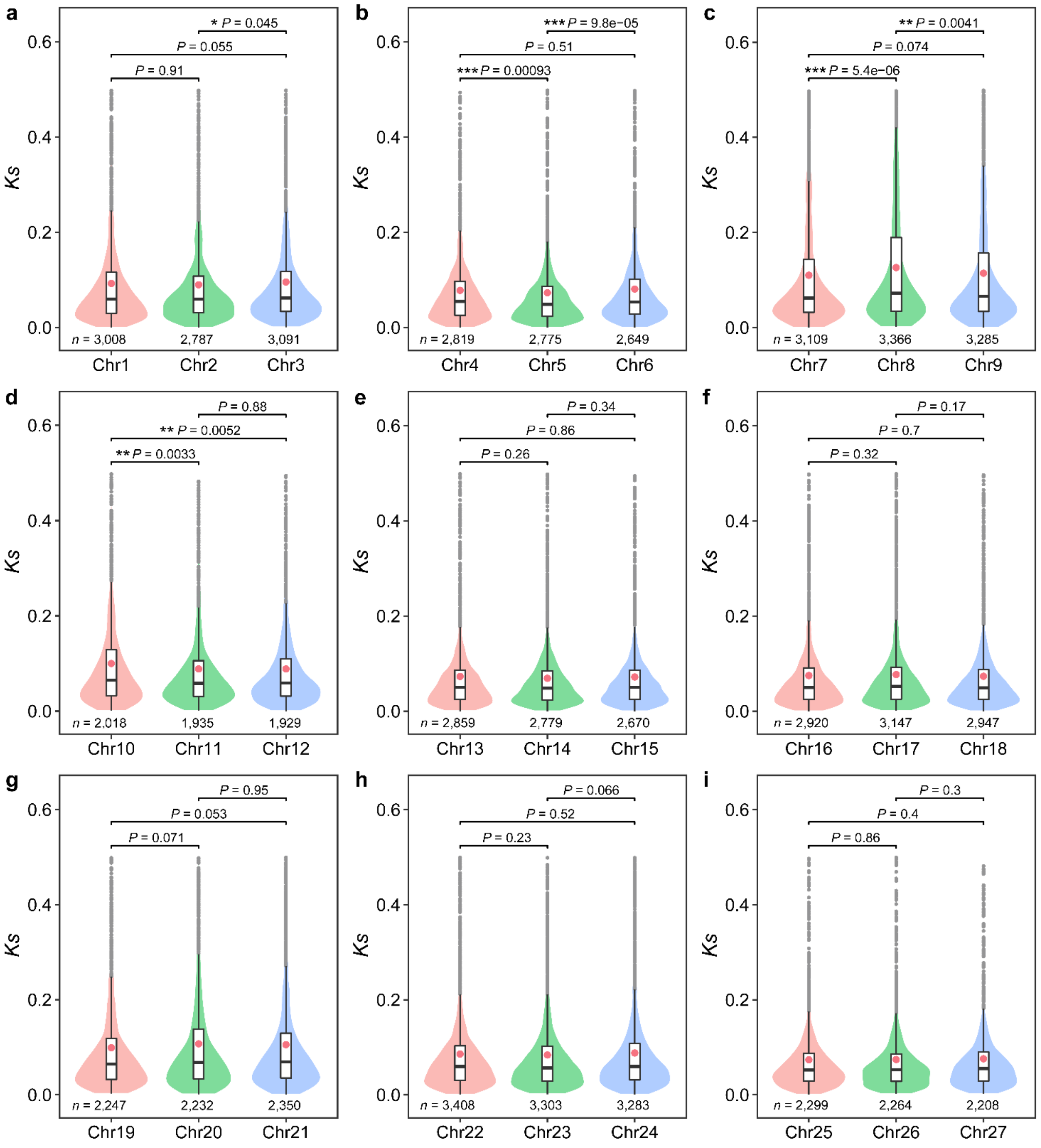

Supplementary Figure 19. *Ks* distribution of orthologous gene pairs between *C. morifolium* and *C. nankingense*. The violin plots show the *Ks* comparison within homoeologous group 1 (**a**) to homoeologous group 9 (**i**) of *C. morifolium*. The central line for each box plot is the median value, the top and bottom edges correspond to the 25th and 75th percentiles, and the whiskers represent the 1.5 times the inter-quartile range (IQR) extending from the edges of the box. The grey points are outliers, and the mean values of each group are indicated by red circles. Numbers of orthologous gene pairs in each panel are indicated as n. Two-tailed Wilcoxon test was used to generate the *P* values (* $P < 0.05$, ** $P < 0.01$, *** $P < 0.001$). Source data are provided as a Source Data file.

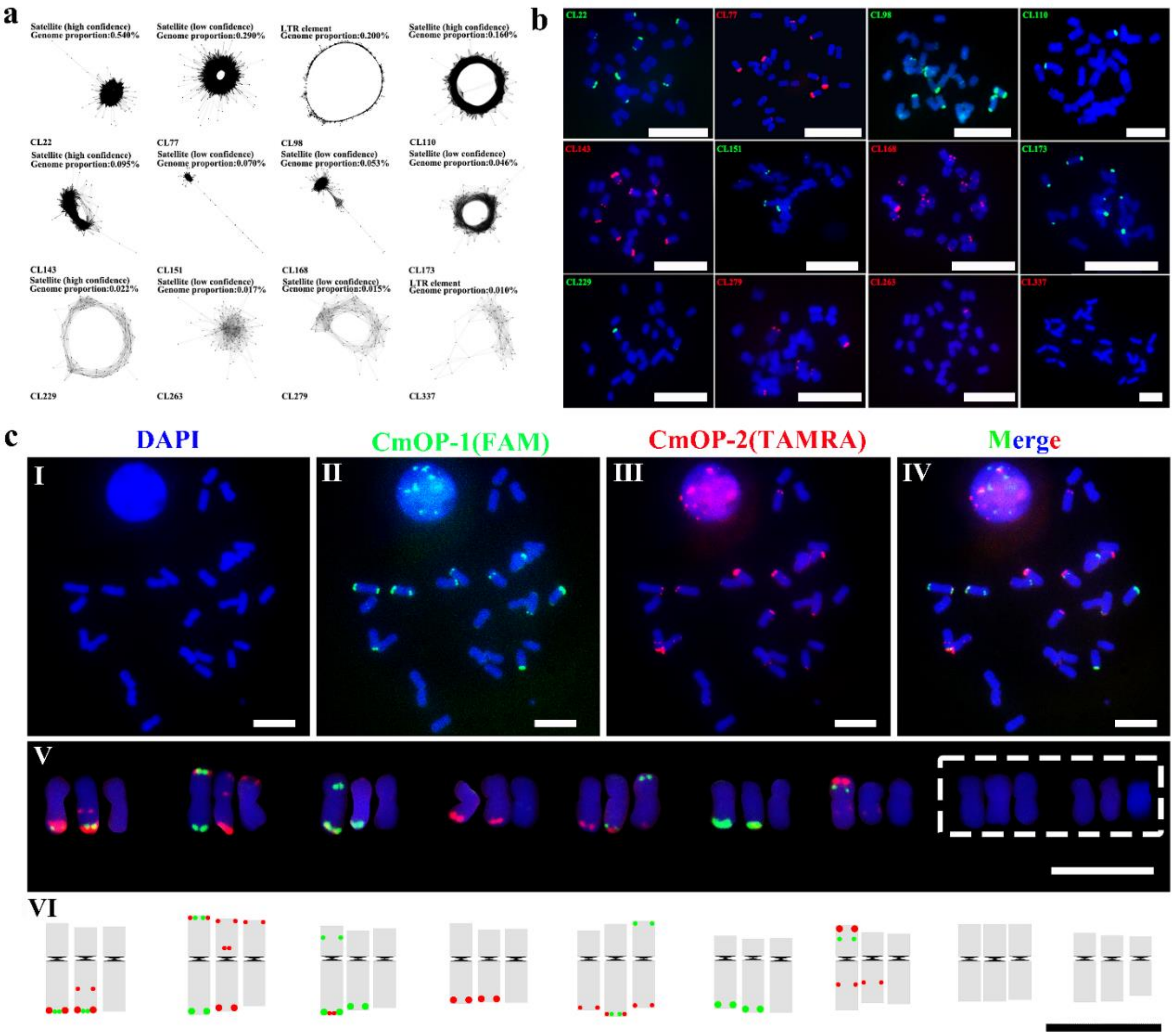

Supplementary Figure 20. RepeatExplore2-TAREAN analysis and Oligo-FISH results of the diploid plant of *C. morifolium* cv. 'Zhongshanzigui'. a Graphic output of RepeatExplore2-TAREAN analysis based on the Illumina reads. **b** Results of the 12 designed probes used for Oligo-FISH in *C. morifolium* (Bar = 10 μm). **c** The Oligo-FISH results by using CmOP-1 (FAM, green) and CmOP-2(TAMRA, red) (Bar = 10 μm). **I** DAPI channel, **II** green channel, **III** red channel, **IV** merge channel, **V** the Oligo-FISH karyotype. White dotted frame shows the two signal-less chromosome groups. **VI** The chromosome idiograms of *C. morifolium*. The signals of CmOP-1 (green) and CmOP-2 (red) are marked in circle (Bar = 10 μm). Each Oligo-FISH experiment in **b** and **c** was independently repeated three slides (30 cells per slide) with 88.9% cells showing similar results. The idiograms in **c** **VI** was drew based on the congruous Oligo-FISH results.

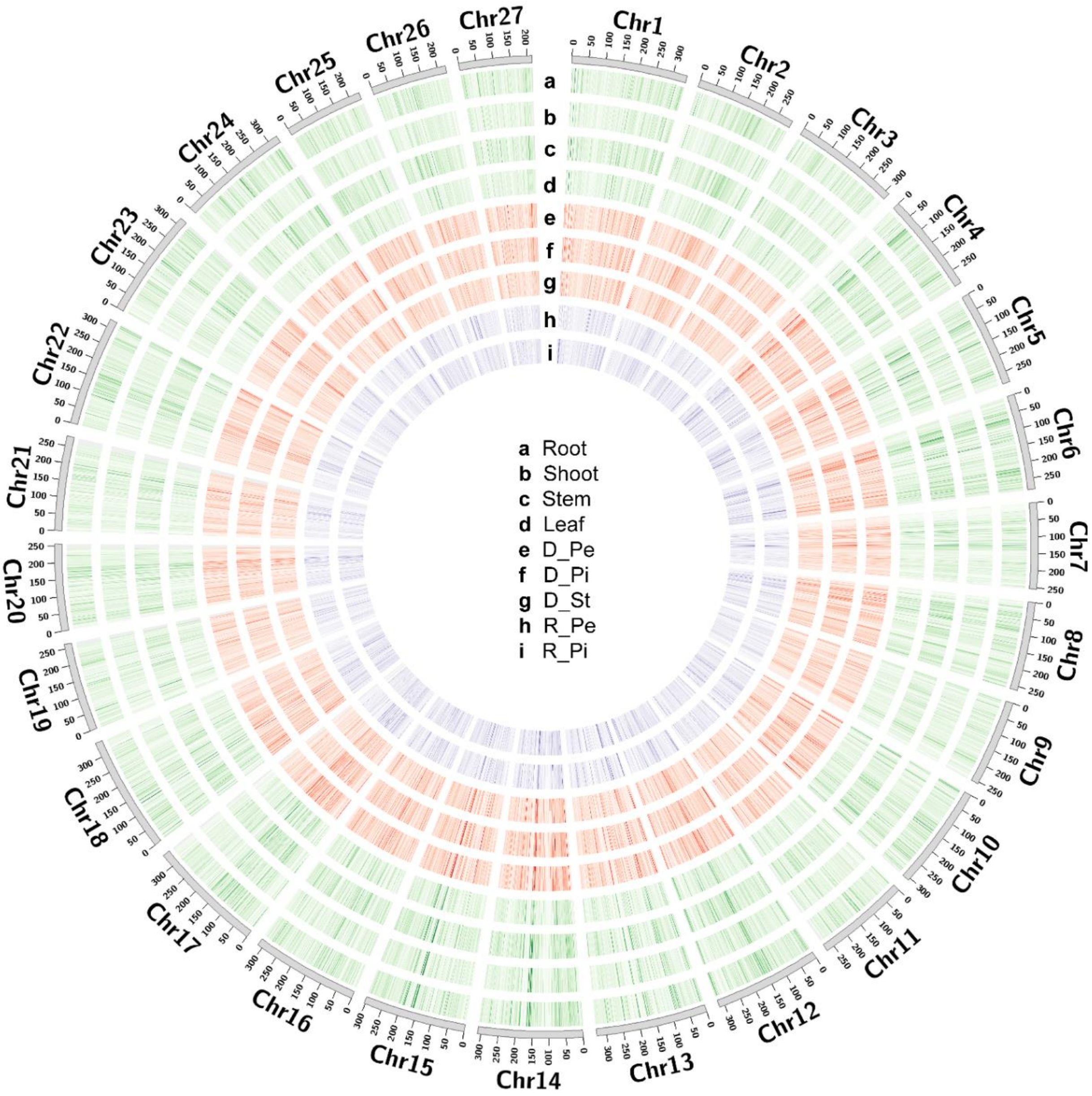

Supplementary Figure 21. Expression levels of syntenic genes within each homoeologous group for nine tissues. Expression levels were calculated based on the average values of nine organ tissues in fragments per kilobase of exon model per million reads mapped (FPKM values). The tracks from outer to inner circles (**a**~**i**) indicate the nine organ tissues. D_Pe, D_Pi, D_St indicate the petals, pistils, stamens of disc florets, respectively; R_Pe and R_Pi indicate the petals and pistils of ray florets, respectively. The RNA sequencing data was obtained from Ding *et al*.[3]. See Supplementary Note 6 for details.

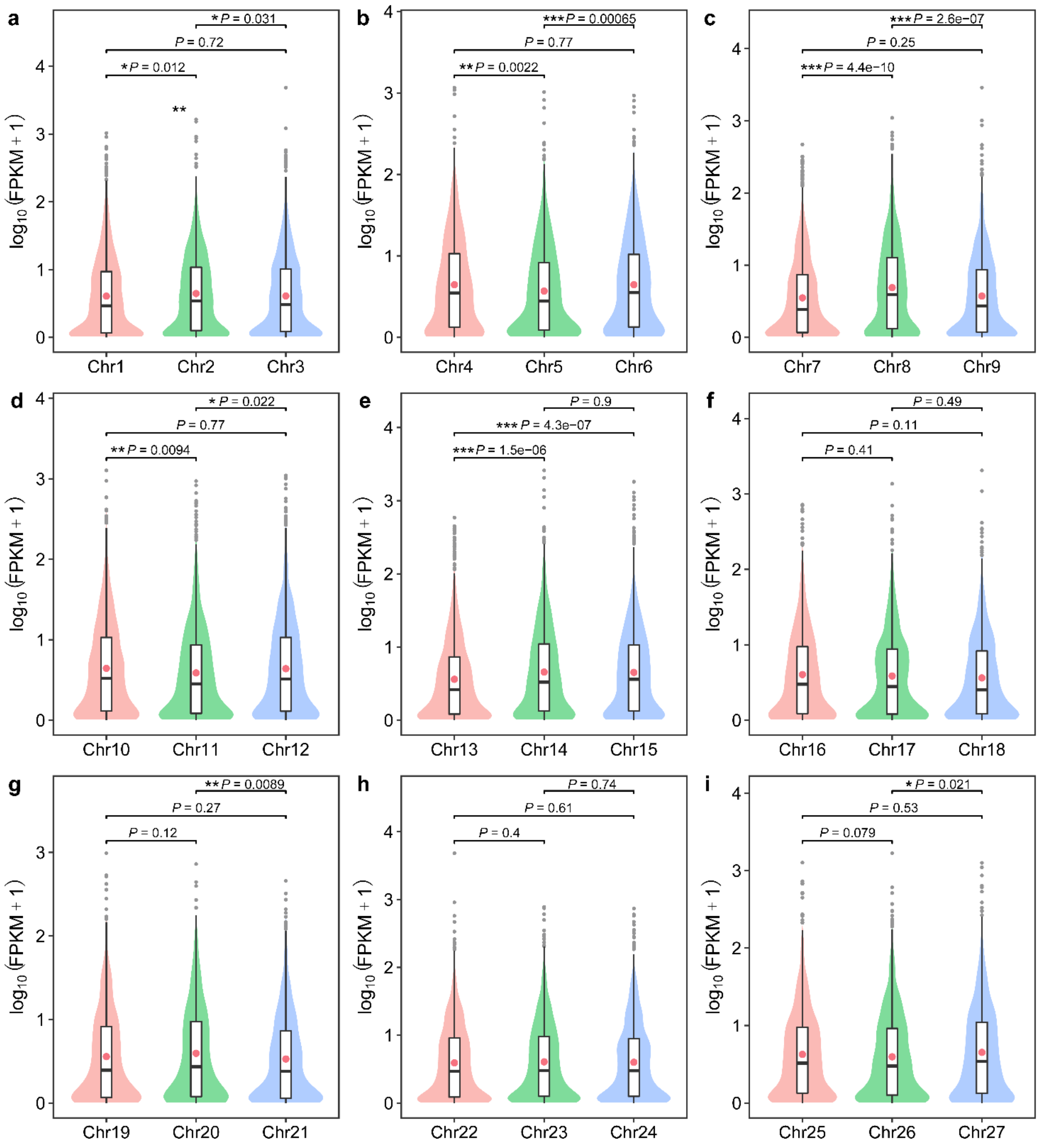

Supplementary Figure 22. Expression levels of syntenic genes within each homoeologous group. Expression levels were calculated based on the average values of nine organ tissues in fragments per kilobase of exon model per million reads mapped (FPKM values). The central line for each box plot is the median value, the top and bottom edges correspond to the 25th and 75th percentiles, and the whiskers represent the 1.5 times the inter-quartile range (IQR) extending from the edges of the box. The grey points are outliers, and the mean values of each group are indicated by red circles (n = 1,826, 1,165, 1,506, 1,435, 1,745, 1,148, 1,046, 1,927, and 1,572 syntenic genes within homoeologous group 1 (**a**) to homoeologous group 9 (**i**), respectively). Two-tailed Wilcoxon test was used to generate the *P* values (* $P < 0.05$, ** $P < 0.01$, *** $P < 0.001$). Source data are provided as a Source Data file.

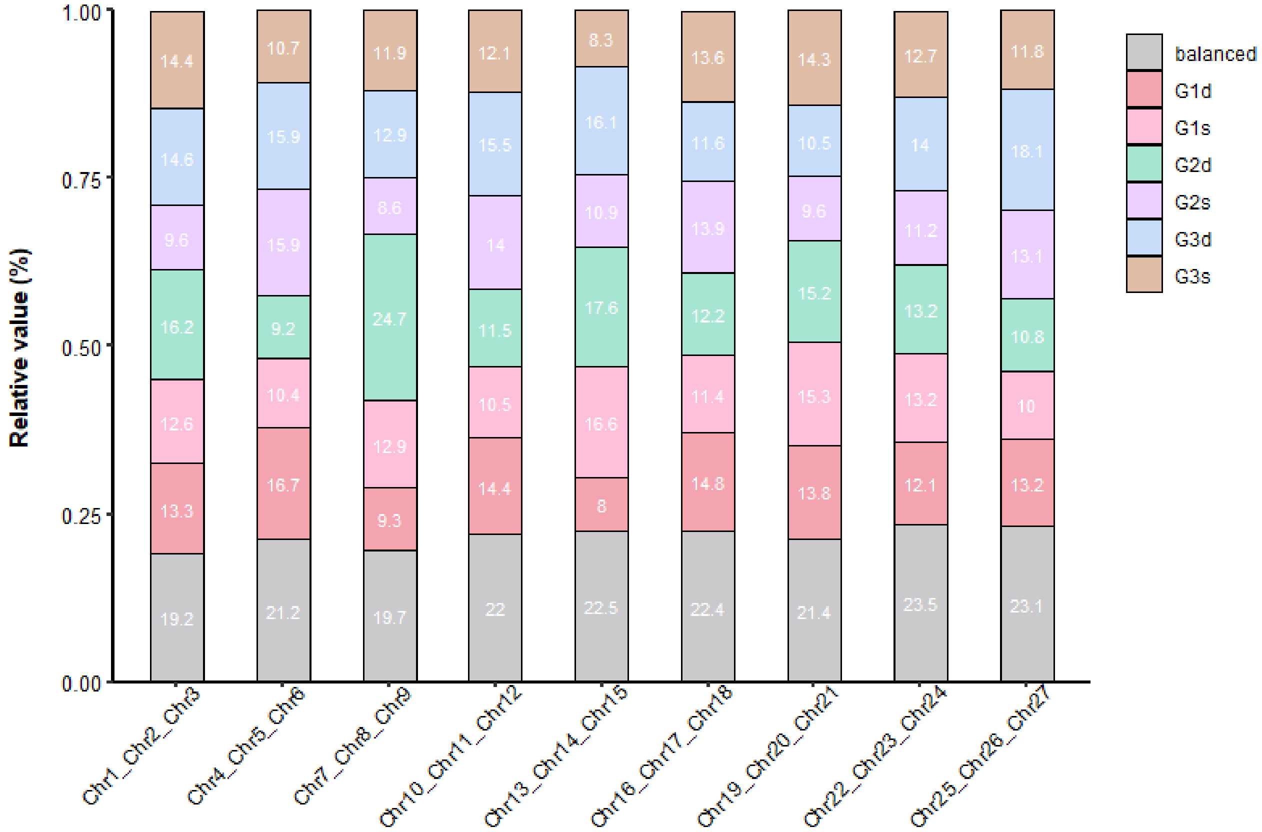

Supplementary Figure 23. Proportion of triads in each category of homoeolog expression bias in *C. morifolium*. The average FPKM values of nine organ tissues were used for the homoeolog expression dominance analysis. G1d, G2d, G3d represent the three dominant categories where the genes located in the first, second, third chromosome has higher abundance of transcripts within a group, respectively. G1s, G2s, G3s represent the three suppressed categories where the genes located in the first, second, third chromosome has lower abundance of transcripts within a group, respectively. The balanced category represents a similar relative abundance of transcripts from the three homoeologs. Source data are provided as a Source Data file.

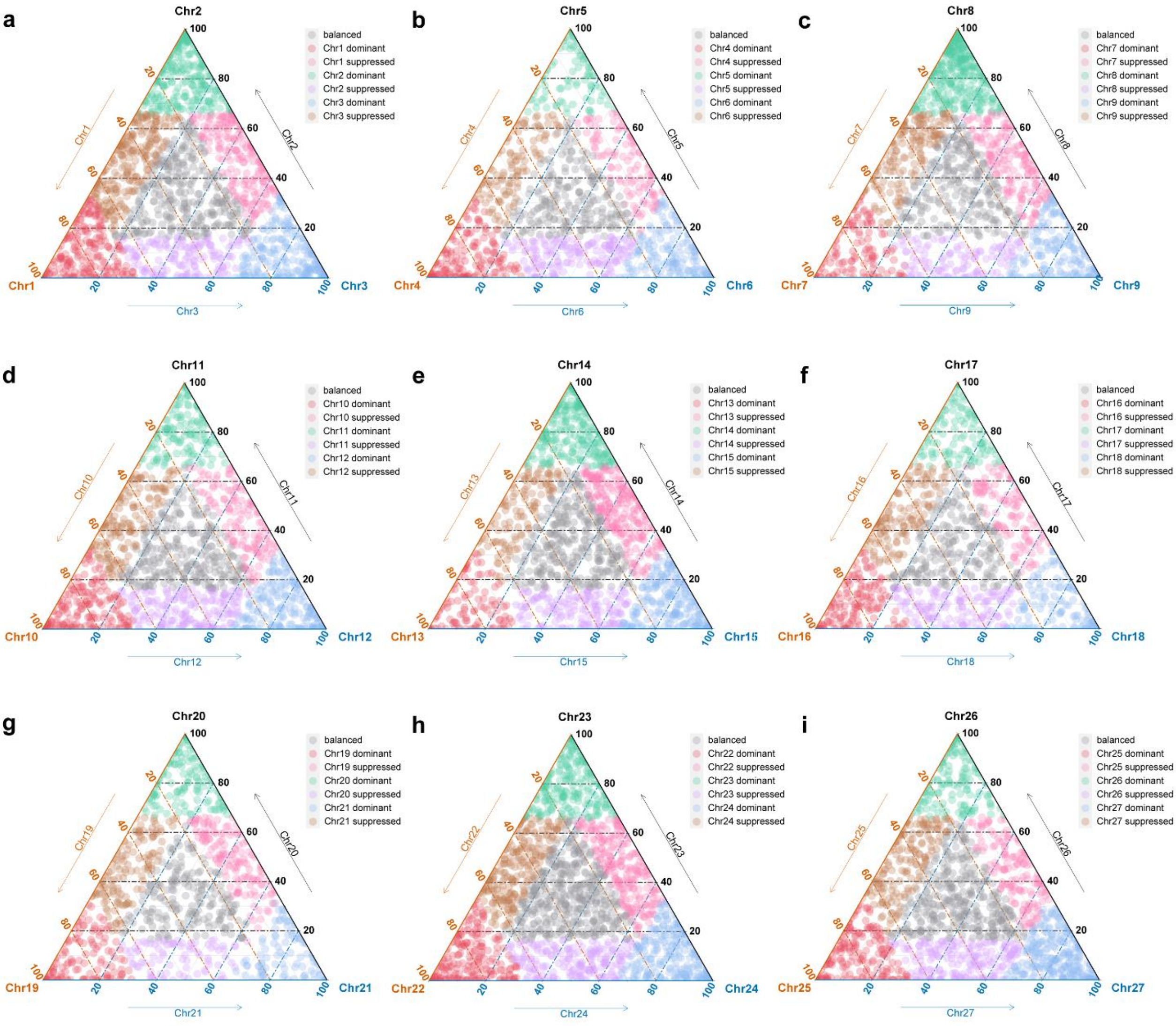

Supplementary Figure 24. Ternary plot showing relative expression abundances of the syntenic triads. The average FPKM values of nine organ tissues were used for the homoeolog expression dominance analysis. Each circle represents a gene triad consisting the syntenic paralogous genes within a given homoeologous group. **a~i** indicate the nine homoeologous groups of *C. morifolium*. Triads in vertices represent the corresponding dominant categories, whereas triads close to edges and between vertices represents suppressed categories. Balanced triads are shown in gray. Source data are provided as a Source Data file.

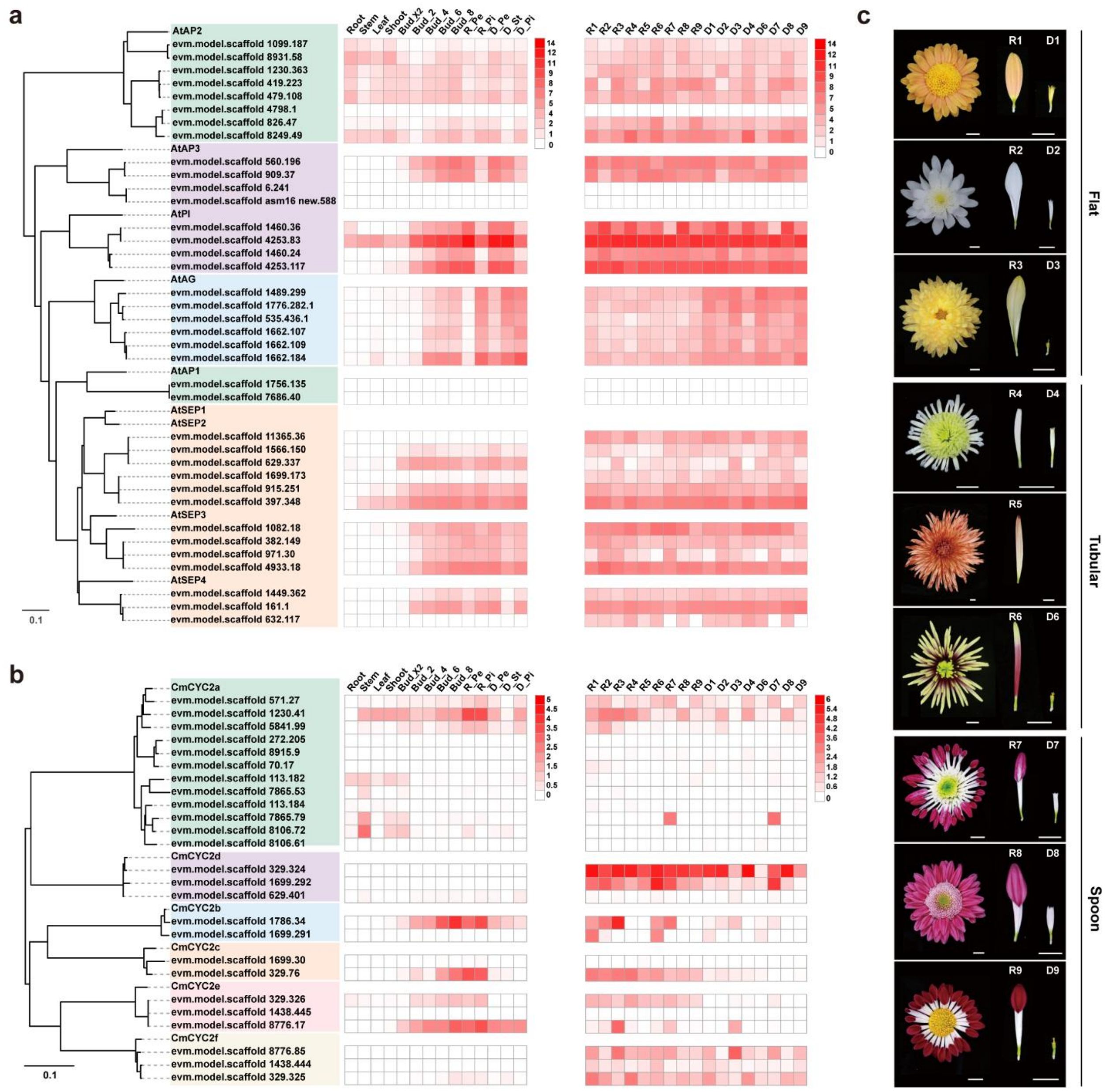

Supplementary Figure 25. The expression patterns of ABCE module genes (a) and *CYC2-like* genes (b) related to flower development. The left heatmap panel in **a** and **b** shows the transcript profiles of different organs from chrysanthemum cultivar ‘Jinba’. Bud_X2, Bud_2, Bud_4, Bud_6, Bud_8 indicate whole buds with diameter < 2 mm, ~2 mm, ~4 mm, ~6 mm, ~8 mm, respectively; D_Pe, D_Pi, D_St indicate the petals, pistils, stamens of disc florets, respectively; R_Pe and R_Pi indicate the petals and pistils of ray florets, respectively. The right heatmap panel in **a** and **b** shows the expression patterns of genes in the ray (R) and discoid (D) florets of three flat petal type cultivars (R/D1~3), three tubular petal type cultivars (R/D4~6), and three spoon petal type cultivars (R/D7~9). The inflorescences of nine cultivars (1~9), i.e., ‘Nannongxixia’, ‘Nannongqingyu’, ‘Qinhuaijinhui’, ‘Nanongxuesong’, ‘Anastasia Brown’, ‘Xuesongyue’, ‘Nannonglifengche’, ‘Nannongziyu’, and ‘Nannongziyunjian’, are denoted in **c**.

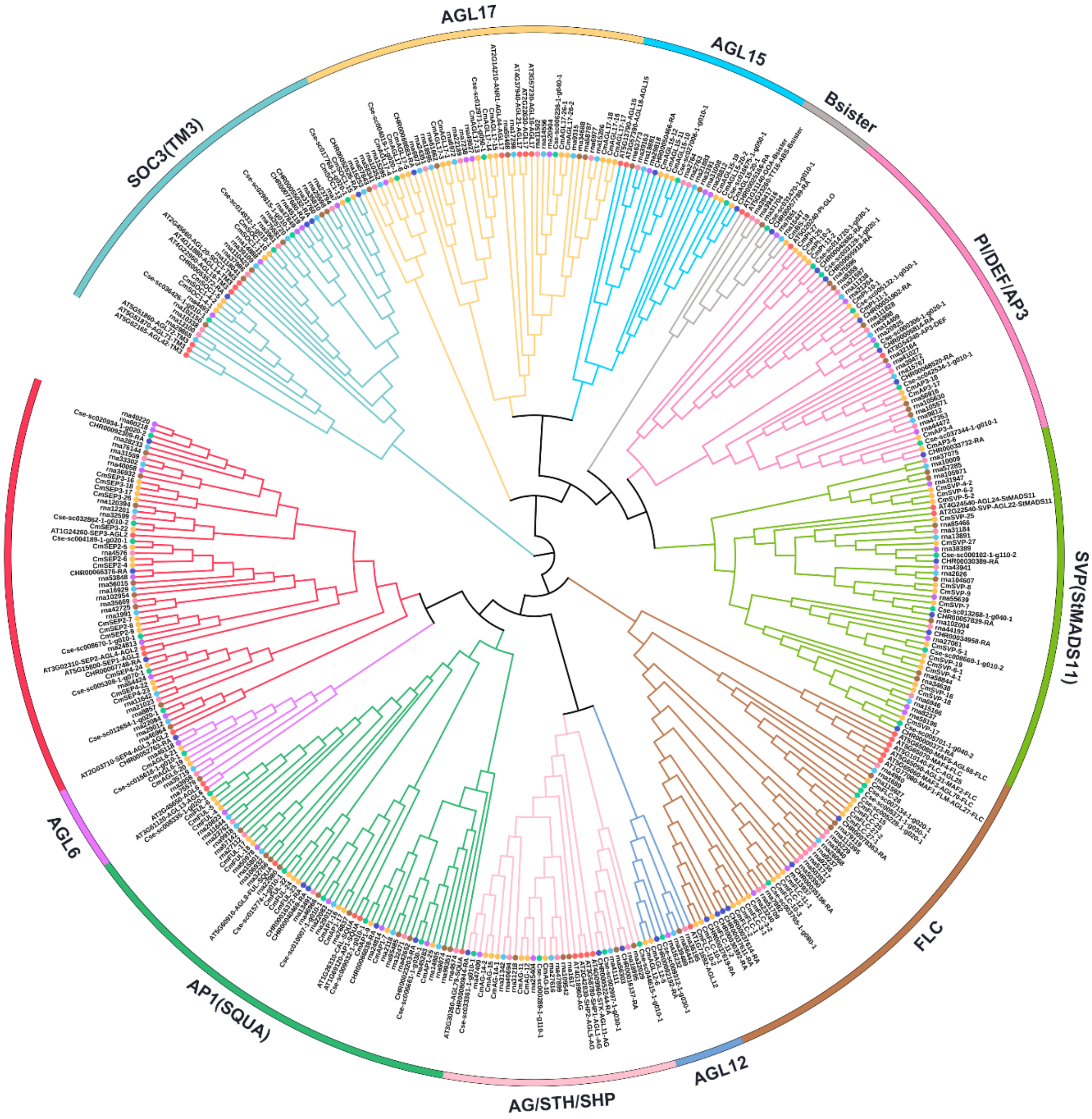

Species	AP1(SQUA)	PI/DEF/AP3	AG/STK/SHP	SEP	Bsister	SOC1 (TM3)	FLC	SVP(StMADS11)	AGL12	AGL17	AGL15	AGL6	Total
Cmo	13	10	6	14	1	7	15	15	2	13	6	3	**105**
Aan	5	4	2	7	1	5	2	6	1	4	3	1	**41**
Ccar	6	4	3	7	1	4	2	4	1	9	2	1	**44**
Cna	4	6	3	3	1	4	6	4	1	1	2	1	**36**
Cse	5	6	4	6	1	4	4	4	1	3	2	1	**41**
Han	10	7	5	8	1	6	3	7	1	5	2	1	**56**
Lsa	5	4	3	5	2	5	10	5	1	5	3	1	**49**
Ath	4	2	4	4	2	6	6	2	1	4	2	2	**39**

Supplementary Figure 26. Phylogeny of MADS-box genes identified in *C. morifolium* and 7 other sequenced plant genomes. The MADS-box proteins of *C. morifolium* are marked by the orange point. Bottom panel shows numbers of the 12 subclades in *C. morifolium* and 7 other sequenced plant genomes. The scientific name of each species is list in Supplementary Data 3.

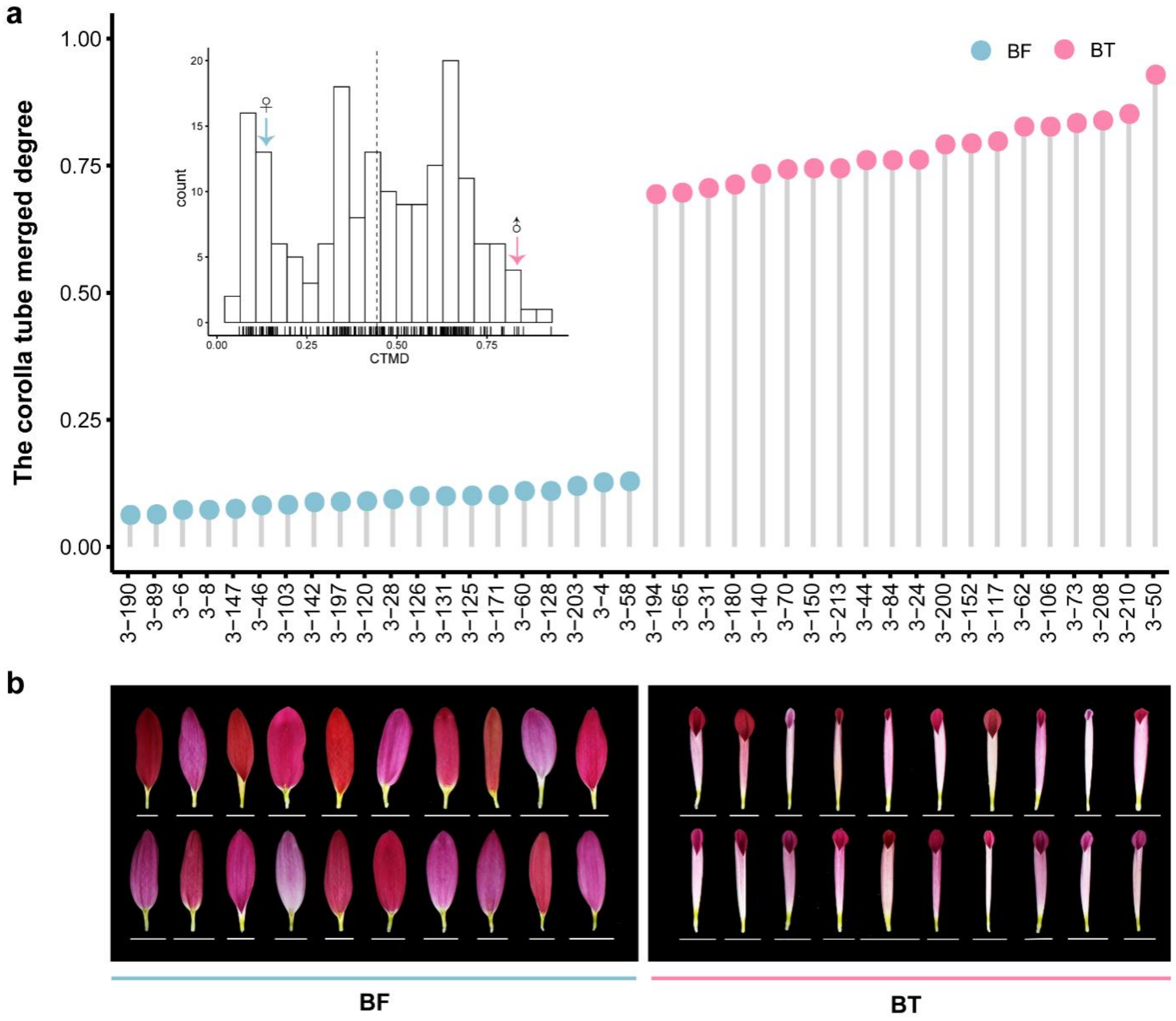

Supplementary Figure 27. BSA-seq analysis of petal shape in chrysanthemum using a F1 population. a Frequency distribution of the corolla tube merged degree and extreme bulk selections. The blue and red arrows indicate the mean phenotypic values of female parent ‘Hongxiao’ (HX) and male parent ‘Q5-12’, respectively. **b** Petal morphology of the 20 selected flat-type progeny (BF) and 20 tubular-type progeny (BT). Bar = 1 cm.

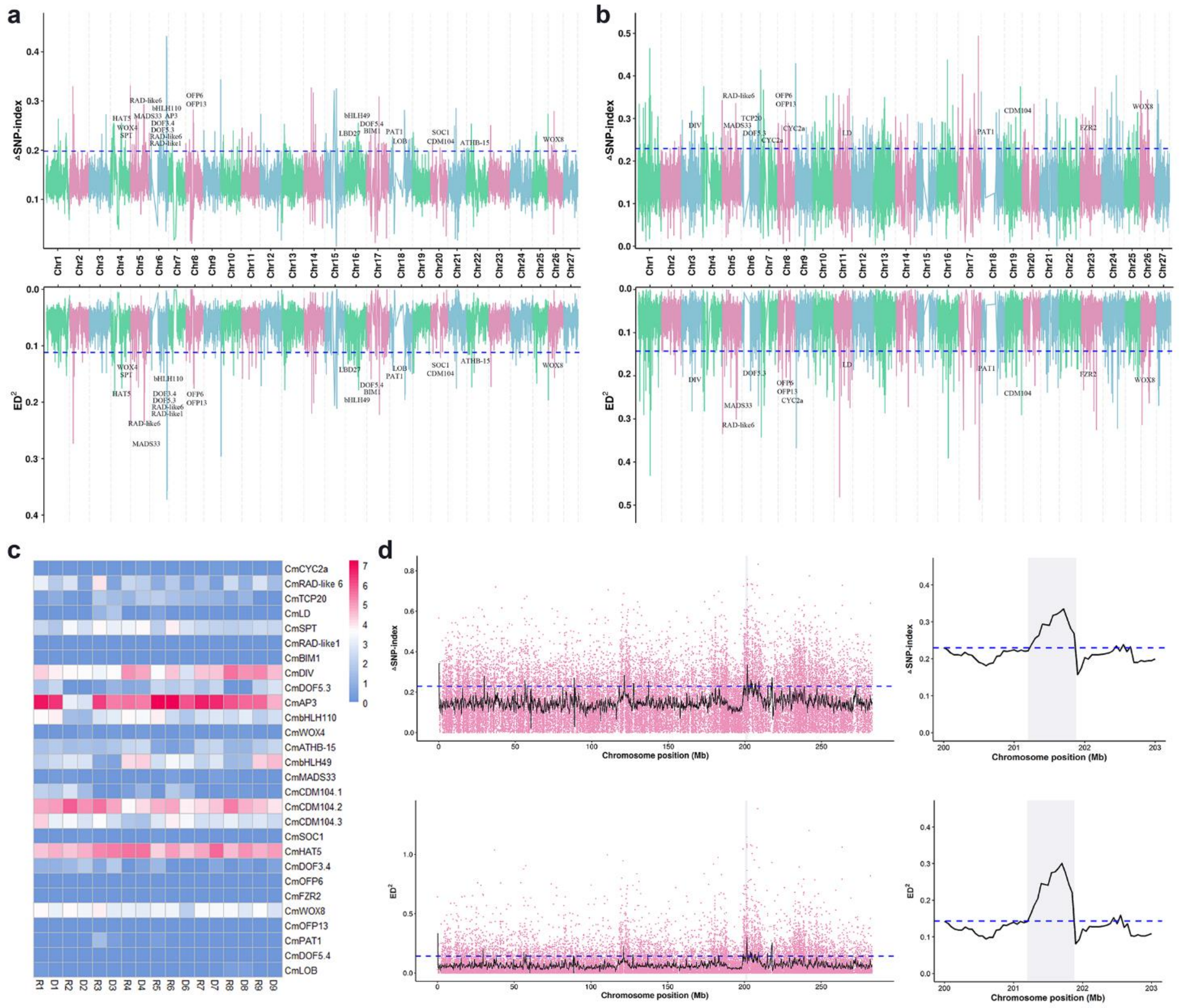

Supplementary Figure 28. Potential chromosomal regions of petal shape loci in chrysanthemum. Distribution of the absolute ΔSNP-index and ED^2 values calculated in a 500 kb window with 50 kb sliding step in chrysanthemum genome using SNPs (**a**) and Indels (**b**). The blue dotted lines indicate the top 1% threshold. **c** The expression patterns of the identified potential candidate genes. **d** Scatter plots for chromosome 5 using Indel markers. *qPT5-5* is highlighted in the right panel with grey shadings.

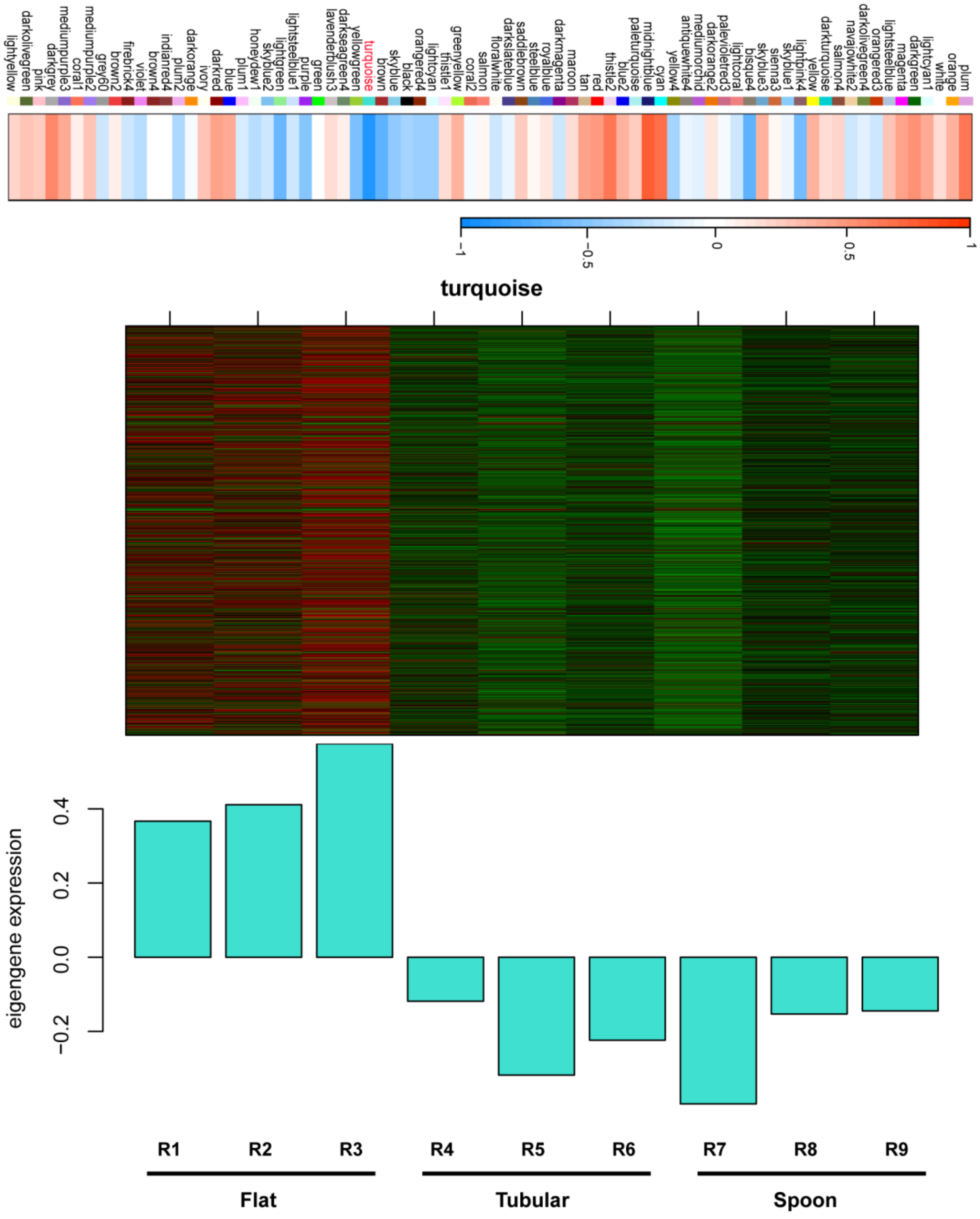

Supplementary Figure 29. WGCNA analysis of petal shape in chrysanthemum. Top panel represents the relationship between each module and corolla tube merged degree. The heatmap and bar graphs of co-expressed genes in turquoise module are shown in bottom panel. R1~R3 represent the ray florets of three flat petal type cultivars. R4~R6 represent the ray florets of three tubular petal type cultivars. R7~R9 represent the ray florets of three spoon petal type cultivars. See Supplementary Note 7 for additional details.

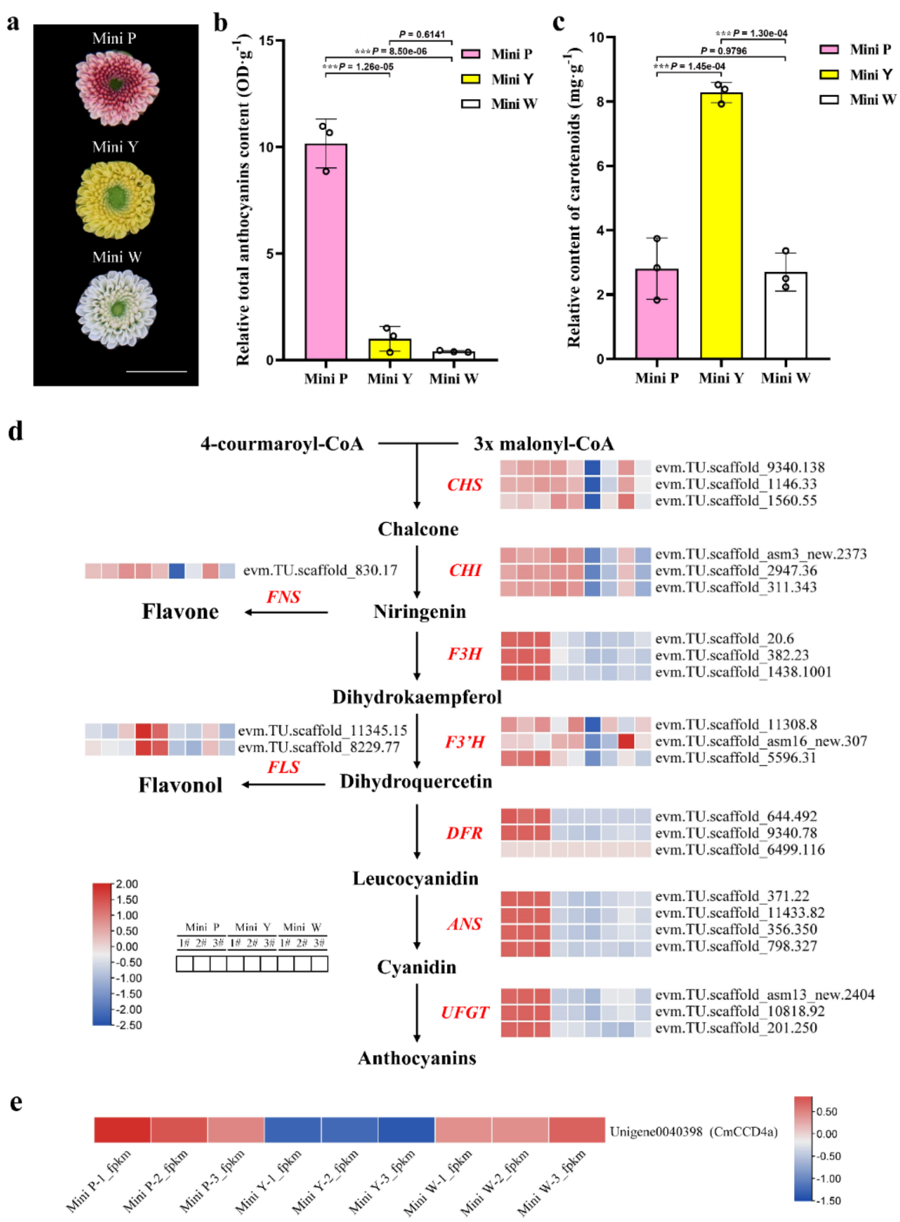

Supplementary Figure 30. The biosynthesis pathways of anthocyanin and flavonol. a Photograph shows the capitulum phenotypes of 'Mini Pink' (Mini P), 'Mini Yellow' (Mini Y) and 'Mini White' (Mini W). Bar = 1 cm. **b** Bar graph shows the relative total content of anthocyanins in the ray floret petals of three Santini chrysanthemum cultivars. Data are presented as mean ± *SD* (standard deviation) values, n = 3 biologically independent replicates. Significant differences are indicated with asterisks (*** $P < 0.001$, one-way ANOVA and two-tailed Tukey's HSD test for multiple comparisons). **c** Bar graph shows the relative content of carotenoids in the ray floret petals of three Santini chrysanthemum cultivars. Data are presented as mean ± *SD* (standard deviation) values, n = 3 biologically independent replicates. Significant differences are indicated with asterisks (*** $P < 0.001$, one-way ANOVA and two-tailed Tukey's HSD test for multiple comparisons). **d** Gene expression profile of biosynthesis genes in flavonoids biosynthesis pathway of petals among three cultivars (from left to right in each heatmap panel are Mini P, Mini Y, Mini W, respectively, with three biological replicates) are presented in the heatmap alongside the gene names. The bar represents the expression level of each gene (z-score). Low to high expression is indicated by a change in colour from blue to red. **e** The FPKM value of Unigene0040398 (*CmCCD4a*) with three biological replicates among three cultivars.

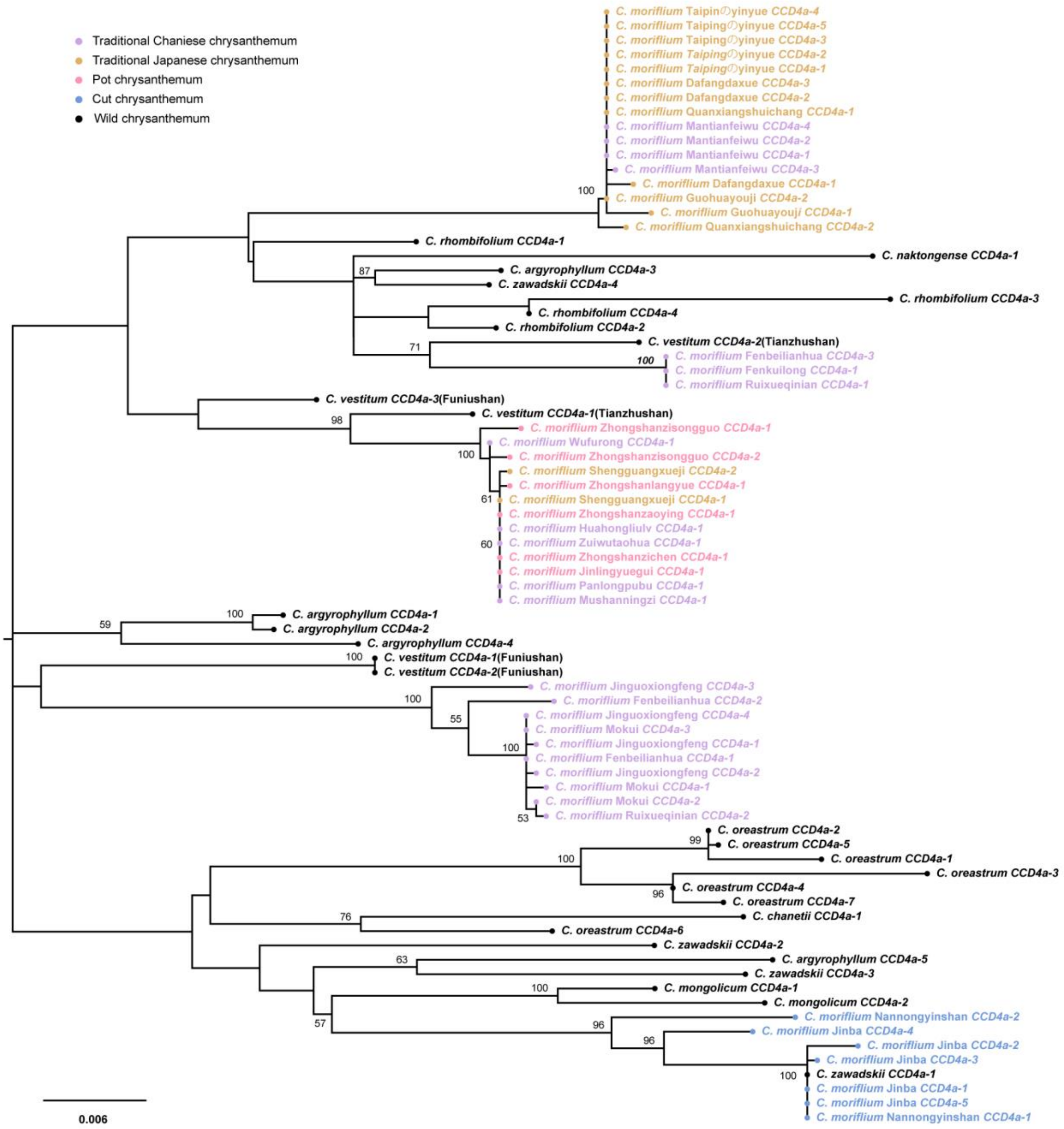

Supplementary Figure 31. Phylogenetic tree of *CCD4a* genes from representative non-yellow wild and cultivated chrysanthemums. The different cultivated types are colour-coded.

Supplementary Table 1. Estimation of genome size of *C. morifolium* using *K*-mer analysis.

Total base (Gb)	*K*-mer	*K*-mer number	*K*-mer depth	Genome size (Mb)	Revised genome size (Mb)	Heterozygous ratio (%)	Repeat ratio (%)
1,070.20	17	332,278,751,852	39	8,519.97	8,469.92	0.71	88.85
1,070.20	21	322,001,348,227	34	9,470.63	8,876.93	0.28	81.75
1,070.20	25	312,032,823,727	33	9,455.54	8,729.39	0.24	77.85
1,070.20	31	297,095,873,649	31	9,583.74	8,744.06	0.16	72.48

Supplementary Table 2. Sequencing libraries and statistics of the data used for *C. morifolium* genome assembly.

Pair-end libraries	Insert size	Total data (G)	Read length (bp)	Sequence coverage (X)
Illumina reads	350 bp	1,070.20	150	126.40
PacBio reads	20 kb	1,022.30	-	120.70
10X Genomics	-	907.50	150	107.20
Hi-C	-	1,002.90	150	118.50
Total	-	4,002.90	-	472.80

Supplementary Table 3. Summary of the final *C. morifolium* genome assembly.

Sample ID	Size (bp)		Number	
	Contig	Scaffold	Contig	Scaffold
Total	8,125,339,779	8,154,322,084	19,524	5,953
Longest	9,959,356	343,668,656	-	-
Number >= 2000 bp	-	-	19,297	5,801
N50/L50	1,867,062	303,688,045	1,311	13
N60/L50	1,465,000	283,376,936	1,808	16
N70/L50	1,128,790	273,155,101	2,439	19
N80/L50	775,922	256,086,517	3,300	22
N90/L50	387,907	232,214,313	4,714	25

Supplementary Table 4. General statistics of the final chromosome-scale *C. morifolium* genome.

Chr	Length (bp)	Contig number	Scaffold number	Gene number	TE content (%)	Gap number	Gap base (N)	N (%)
Chr1	341,398,601	391	205	5,670	84.14	390	1,204,700	0.35
Chr2	295,030,345	429	245	5,202	83.43	428	1,153,022	0.39
Chr3	313,095,894	386	225	5,689	83.58	385	1,007,056	0.32
Chr4	294,124,624	507	394	4,984	84.40	506	688,181	0.23
Chr5	283,376,936	318	178	4,588	84.32	317	867,409	0.31
Chr6	282,905,081	502	207	4,501	79.79	501	693,468	0.25
Chr7	254,060,111	487	218	4,811	81.51	486	686,741	0.27
Chr8	262,101,579	506	343	4,867	84.39	505	951,924	0.36
Chr9	256,086,517	479	307	4,846	84.25	478	982,394	0.38
Chr10	308,229,016	407	243	4,711	85.20	406	968,682	0.31
Chr11	273,155,101	371	203	4,498	84.04	370	1,038,521	0.38
Chr12	316,167,799	661	452	4,714	85.09	660	1,231,094	0.39
Chr13	329,179,289	413	261	5,228	84.87	412	897,466	0.27
Chr14	309,650,168	525	364	5,212	84.58	524	945,337	0.31
Chr15	303,688,045	477	147	5,047	82.27	476	1,044,951	0.34
Chr16	315,184,784	519	308	5,419	83.84	518	1,324,994	0.42
Chr17	340,404,798	804	663	5,899	83.97	803	890,993	0.26
Chr18	343,668,656	815	192	5,275	77.55	814	864,556	0.25
Chr19	263,614,180	463	306	4,730	83.13	462	935,774	0.35
Chr20	255,661,353	582	194	4,734	81.27	581	1,017,174	0.40
Chr21	279,095,745	647	492	4,983	83.41	646	939,585	0.34
Chr22	323,725,715	561	355	5,656	82.85	560	1,308,492	0.40
Chr23	320,737,027	393	229	5,645	83.53	392	1,002,780	0.31
Chr24	329,610,984	470	295	5,830	84.02	469	1,150,356	0.35
Chr25	232,214,313	394	240	4,003	84.31	393	964,868	0.42
Chr26	224,835,988	370	204	3,848	84.25	369	983,054	0.44
Chr27	214,507,245	386	251	3,860	84.06	385	859,910	0.40
Chr0	288,812,190	6,261	5,630	4,299	82.96	320	2,378,823	0.82
Total	8,154,322,084	19,524	13,351	138,749	83.92	13,556	28,982,305	0.36

Supplementary Table 5. Assessment of *C. morifolium* genome using EST sequences.

Dataset	Number	with >90% sequence in one scaffold		with >50% sequence in one scaffold	
		Number	Percentage (%)	Number	Percentage (%)
>0 bp	105,996	99,245	93.63	102,209	96.43
>200 bp	105,844	99,102	93.63	102,058	96.42
>500 bp	24,199	23,314	96.34	24,034	99.32
>1000 bp	8,031	7,810	97.25	8,011	99.75
>2000 bp	1,691	1,638	96.87	1,688	99.82
>5000 bp	42	40	95.24	42	100.00

Supplementary Table 6. Summary of BUSCO and CEGMA evaluations for the chromosome-scale genome assembly of *C. morifolium*.

BUSCO notation	Number	Percentage (%)
Complete	1577	97.70
Complete and single-copy	161	10.00
Complete and duplicated	1416	87.70
Fragmented	3	0.20
Missing	34	2.10
Total BUSCO groups searched	1614	-
**CEGMA notation**	**Number**	**Completeness (%)**
Complete	240	96.77
Complete + partial	244	98.39

Supplementary Table 7. Statistics of the Illumina paired-end sequencing reads mapped to *C. morifolium* genome.

Taxa	Percentage
Mapping rate (%)	98.81
Average sequencing depth (x)	43.11
Coverage (%)	99.2
Coverage at least 4x (%)	98.7
Coverage at least 10x (%)	97.76
Coverage at least 20x (%)	94.98
All SNP (%)	0.0143
Heterozygous SNP (%)	0.0086
homozygous SNP (%)	0.0057

Supplementary Table 8. List of tissues and reads for transcriptome sequencing mapped to *C. morifolium* genome.

Issues	Total reads	Total base pairs (bp)	Total unmapped (%)	Total mapped (%)	Unique mapped (%)	Multi-mapped (%)
D_Pe1	55,688,808	8,353,321,200	17.38	82.62	59.95	22.67
D_Pe2	51,610,636	7,741,595,400	17.90	82.10	59.30	22.80
D_Pe3	56,008,190	8,401,228,500	16.88	83.12	60.08	23.04
D_Pi1	47,576,914	7,136,537,100	18.02	81.98	60.19	21.79
D_Pi2	51,741,526	7,761,228,900	17.87	82.13	58.95	23.18
D_Pi3	52,009,282	7,801,392,300	17.77	82.23	59.34	22.89
D_St1	49,404,564	7,410,684,600	16.84	83.16	58.82	24.35
D_St2	51,640,370	7,746,055,500	17.36	82.64	59.13	23.52
D_St3	55,103,014	8,265,452,100	16.98	83.02	59.17	23.85
Leaf_1	57,805,740	8,670,861,000	16.01	83.99	60.67	23.32
Leaf_2	48,242,218	7,236,332,700	16.44	83.56	60.76	22.81
Leaf_3	45,385,158	6,807,773,700	16.76	83.24	59.41	23.83
Root_1	42,906,370	6,435,955,500	19.07	80.93	58.89	22.04
Root_2	49,965,384	7,494,807,600	19.04	80.96	58.98	21.98
Root_3	66,049,230	9,907,384,500	18.74	81.26	59.51	21.75
R_Pe1	55,441,706	8,316,255,900	16.81	83.19	60.42	22.77
R_Pe2	50,979,492	7,646,923,800	16.56	83.44	60.57	22.87
R_Pe3	59,244,284	8,886,642,600	16.86	83.14	60.51	22.63
R_Pi1	54,049,188	8,107,378,200	17.93	82.07	59.45	22.62
R_Pi2	54,044,472	8,106,670,800	18.23	81.77	59.17	22.60
R_Pi3	55,103,230	8,265,484,500	18.07	81.93	59.32	22.61
Shoot_1	37,688,970	5,653,345,500	19.81	80.19	58.81	21.38
Shoot_2	41,174,000	6,176,100,000	19.52	80.48	58.90	21.58
Shoot_3	61,404,200	9,210,630,000	18.84	81.16	59.38	21.78
Stem_1	43,282,098	6,492,314,700	19.82	80.18	58.31	21.87
Stem_2	54,461,794	8,169,269,100	18.04	81.96	59.42	22.54
Stem_3	47,645,332	7,146,799,800	18.43	81.57	58.90	22.67
Bud_2_1	55,147,492	8,272,123,800	16.86	83.14	59.42	23.72
Bud_2_2	59,669,252	8,950,387,800	16.65	83.35	58.66	24.68
Bud_2_3	58,136,840	8,720,526,000	16.89	83.11	59.52	23.59
Bud_4_1	50,467,038	7,570,055,700	17.39	82.61	59.06	23.55
Bud_4_2	52,972,526	7,945,878,900	17.39	82.61	58.42	24.18
Bud_4_3	60,919,672	9,137,950,800	16.62	83.38	59.23	24.15
Bud_6_1	57,651,800	8,647,770,000	17.80	82.20	59.98	22.22
Bud_6_2	56,258,850	8,438,827,500	17.36	82.64	59.07	23.57
Bud_6_3	53,001,398	7,950,209,700	17.05	82.95	60.06	22.89
Bud_8_1	56,999,034	8,549,855,100	18.87	81.13	59.36	21.77
Bud_8_2	56,139,374	8,420,906,100	18.51	81.49	59.82	21.67
Bud_8_3	51,193,506	7,679,025,900	17.65	82.35	59.33	23.02

Bud_X2_1	53,325,910	7,998,886,500	17.54	82.46	59.41	23.05
Bud_X2_2	53,998,800	8,099,820,000	17.37	82.63	59.68	22.95
Bud_X2_3	63,674,144	9,551,121,600	16.90	83.10	59.28	23.83
Total	2,235,211,806	335,281,770,900	17.69	82.31	59.44	22.87

Note: Bud_X2, Bud_2, Bud_4, Bud_6, Bud_8 indicate whole buds with diameter <2 mm, ~2 mm, ~4 mm, ~6 mm, ~8 mm, respectively; D_Pe, D_Pi, D_St indicate the petals, pistils, stamens of disc florets, respectively; R_Pe and R_Pi indicate the petals and pistils of ray florets, respectively.

Supplementary Table 9. Sequencing libraries and statistics of the data used for *C. nankingense* genome assembly.

Pair-end libraries	Insert size	Total data (G)	Read length (bp)	Sequence coverage (X)
Illumina reads	400	149.90	150	48.51
PacBio reads	-	355.50	30870 (N50)	115.05
HiC	350	566.68	150	183.39
Total	-	1,072.08	-	346.95

Supplementary Table 10. General statistics of the final chromosome-scale *C. nankingense* genome.

Chr	Length (bp)	Contig number	Gene number	Gap base (N)	N (%)
Cna1	378,326,653	447	4,779	44,600	0.0118
Cna2	308,287,845	377	3,987	37,600	0.0122
Cna3	253,387,367	364	3,927	36,300	0.0143
Cna4	260,249,678	336	3,002	33,500	0.0129
Cna5	431,328,461	918	4,788	91,700	0.0213
Cna6	377,401,742	400	4,837	39,900	0.0106
Cna7	300,629,015	309	4,301	30,800	0.0102
Cna8	353,782,469	463	5,190	46,200	0.0131
Cna9	211,314,829	375	3,184	37,400	0.0177
Total	2,874,708,059	3,989	37,995	398,000	0.1240

Supplementary Table 11. Functional annotation of the predicted protein-coding genes for *C. morifolium*.

	Database	Number	Percentage (%)
Annotated	NR	126,222	90.97
	Swiss-Prot	104,862	75.58
	GO	125,952	90.78
	KEGG	97,516	70.28
	InterPro	137,021	98.75
	Pfam	100,735	72.60
	Total	137,820	99.33
Unannotated		929	0.67
Total		138,749	-

Supplementary Table 12. Annotation of non-coding RNA genes in the *C. morifolium* genome.

Type		Number	Average length (bp)	Total length (bp)	% of genome
miRNA		2,280	113.29	258,301	0.0032
tRNA		4,102	74.28	304,712	0.0037
rRNA	18S	419	1,412.85	591,985	0.0073
	28S	1,341	143.23	192,076	0.0024
	5.8S	334	159.05	53,123	0.0007
	5S	774	116.60	90,250	0.0011
	Total	2,868	323.37	927,434	0.0114
snRNA	CD-box	39,924	107.01	4,272,177	0.0524
	HACA-box	222	131.73	29,244	0.0004
	splicing	1,452	140.50	203,999	0.0025
	Total	41,598	108.31	4,505,675	0.0553

Supplementary Table 13. Mapping summary of transcriptome from multiple tissues to the *C. morifolium* genes.

Issue	Expressed gene	Unexpressed gene	Genome coverage (%)
Leaf	76,690	62,059	50.73
Root	79,613	59,136	54.23
Stem	77,603	61,146	52.23
Shoot	78,414	60,335	52.81
Bud_X2	80,244	58,505	57.28
Bud_2	78,538	60,211	58.13
Bud_4	78,296	60,453	57.37
Bud_6	80,285	58,464	59.64
Bud_8	79,469	59,280	56.69
D_Pe	82,320	56,429	59.74
D_Pi	80,932	57,817	59.71
D_St	84,734	54,015	72.33
R_Pe	74,937	63,812	52.35
R_Pi	79,288	59,461	59.35
Total	103,287	35,462	--

The raw reads were download from National Center for Biotechnology Information with the BioProject ID PRJNA548460.

Supplementary Table 14. Summary of repetitive element contents in *C. morifolium* genome.

Class	Number	Length (bp)	% in genome	% in TE	Mean length (bp)
SINE	12,765	9,492,825	0.116	0.140	743.66
LINE	367,556	219,221,060	2.688	3.224	596.43
LTR/*Copia*	4,426,916	3,294,752,218	40.405	48.461	744.25
LTR/*Gypsy*	2,111,885	2,021,624,392	24.792	29.735	957.26
LTR/other	768,516	944,604,703	11.584	13.894	1,229.13
DNA	879,220	703,917,330	8.632	10.354	800.62
Simple repeat	39,888	61,905,536	0.759	0.911	1,551.98
Satellite	12,662	13,284,107	0.163	0.195	1,049.13
Other	19	49,821	0.001	0.001	2,622.16
Total	8,779,048	6,798,757,725	83.376	100.000	774.43

Supplementary Table 15. The statistics of synteny genes in *C. morifolium* genome.

Chr	Gene number	Synteny gene number	Synteny gene order consistent	Synteny gene percentage (%)	Synteny gene order consistent percentage (%)
Chr1	5,670	1,826	1,455	32.20	79.68
Chr2	5,202	1,826	1,455	35.10	79.68
Chr3	5,689	1,826	1,455	32.10	79.68
Chr4	4,984	1,633	1,486	32.76	91.00
Chr5	4,588	1,633	1,486	35.59	91.00
Chr6	4,501	1,633	1,486	36.28	91.00
Chr7	4,811	1,787	1,660	37.14	92.89
Chr8	4,867	1,787	1,660	36.72	92.89
Chr9	4,846	1,787	1,660	36.88	92.89
Chr10	4,711	1,435	1,206	30.46	84.04
Chr11	4,498	1,435	1,206	31.90	84.04
Chr12	4,714	1,435	1,206	30.44	84.04
Chr13	5,228	2,006	1,781	38.37	88.78
Chr14	5,212	2,006	1,781	38.49	88.78
Chr15	5,047	2,006	1,781	39.75	88.78
Chr16	5,419	1,789	1,446	33.01	80.83
Chr17	5,899	1,789	1,446	30.33	80.83
Chr18	5,275	1,789	1,446	33.91	80.83
Chr19	4,730	1,262	1,194	26.68	94.61
Chr20	4,734	1,262	1,194	26.66	94.61
Chr21	4,983	1,262	1,194	25.33	94.61
Chr22	5,656	1,927	1,649	34.07	85.57
Chr23	5,645	1,927	1,649	34.14	85.57
Chr24	5,830	1,927	1,649	33.05	85.57
Chr25	4,003	1,572	1,334	39.27	84.86
Chr26	3,848	1,572	1,334	40.85	84.86
Chr27	3,860	1,572	1,334	40.73	84.86

Supplementary Table 16. The correlation between gene orientation change and gene expression change.

Group	Correlation coefficient	*P* value
Chr1-Chr2-Chr3	-0.082	1.14E-03
Chr4-Chr5-Chr6	-0.017	5.77E-01
Chr7-Chr8-Chr9	-0.051	6.48E-02
Chr10-Chr11-Chr12	-0.042	1.41E-01
Chr13-Chr14-Chr15	-0.043	1.10E-01
Chr16-Chr17-Chr18	0.055	8.42E-02
Chr19-Chr20-Chr21	-0.020	5.50E-01
Chr22-Chr23-Chr24	0.020	4.05E-01

Supplementary Note 1. Genome sequencing and survey of *C. morifolium*

Illumina library preparation

Sequencing libraries with insert sizes of 350 bp were constructed using a library construction kit according to the manufacturer's instructions (Illumina, San Diego, CA). These libraries were then sequenced using an Illumina HiSeq X platform.

PacBio library construction and sequencing

For 20-kb-insert-size library construction, at least 10 μg of sheared DNA is required. SMRTbell template preparation involved DNA concentration, damage repair, end repair, hairpin adapter ligation, and template purification. Finally, we carried out 20 kb single-molecule real-time (SMRT) DNA sequencing by PacBio and sequenced the DNA library on the PacBio Sequel platform.

10X Genomics library construction and sequencing

DNA sample preparation, indexing, and barcoding were carried out using a GemCode Instrument from 10X Genomics. A DNA sample of 1 ng with a length of 50 kb was used for the GEM reaction procedure during PCR, and 16 bp barcodes were introduced into droplets. Then, the droplets were fractured following the purification of the intermediate DNA library. The library was finally sequenced on an Illumina HiSeq X instrument.

Hi-C library construction and sequencing

DNA from young leaves of the same *C. morifolium* plant was used as starting material for the high throughput chromatin conformation capture (Hi-C) library. Formaldehyde was used for fixing chromatin. The leaf cells were lysed and *Hind*III endonuclease was used for digesting the fixed chromatin. The 5' overhangs of the DNA were recovered with biotin-labeled nucleotides and the resulting blunt ends were ligated to each other using DNA ligase. Proteins were removed with protease to release the DNA molecules from the crosslinks. The purifified DNA was sheared into 350 bp fragments and ligated to adaptors[4]. The fragments labeled with biotin were extracted using streptavidin beads and after PCR enrichment, the libraries were sequenced on Illumina HiSeq X instrument.

RNA library preparation and sequencing

Total RNA was extracted from the different types of organs using an RNAprep Pure Plant Kit (TIANGEN, Beijing, China), and genomic DNA contaminants were removed using RNase-Free DNase I (TIANGEN, Beijing, China). The integrity of RNA was evaluated on a 1.0% agarose gel stained with ethidium bromide (EB), and its quality and quantity were assessed by using an Agilent 2100 Bioanalyzer (Agilent Technologies, CA, USA). Then, the integrated RNA was used for cDNA library construction and Illumina sequencing. The cDNA library was constructed using an NEBNext Ultra RNA Library Prep Kit for Illumina (NEB) following the manufacturer's recommendations.

Prepared libraries were sequenced on the Illumina HiSeq X platform, generating 150 bp PE reads.

Genome survey

Genome characteristics was estimated by analysing *K*-mer frequency using Illumina sequence data[5]. The genome size of the haploid line of *C. morifolium* cv. 'Zhongshanzigui' was estimated based on *K*-mer (K = 17, 21, 25 and 31) statistics, using the modified Lander-Waterman algorithm:

$$G = (N \times (L - K + 1) - B)/D \qquad (\mathbf{1})$$

where G is the genome size, N is the total number of sequence reads, L is the average length of sequence reads, K is the *K*-mer length (bp)[6], B is the total number of low-frequency *K*-mers (frequency ≤ 1 in this analysis), and D is the overall depth. Heterozygosity was reflected by distributions of the number of distinct *K*-mers.

Supplementary Note 2. Chromosomal assembly of *C. nankingense*

Genome assembly and annotation

The diploid *C. nankingense* genome was assembled using a comprehensive dataset by incorporating Illumina short reads, PacBio SMRT long-reads as well as high throughput chromatin conformation capture (Hi-C) data. A total of 360 Gb (~120× coverage) subreads were generated from the PacBio Sequel II platform. All of the subreads were corrected, trimmed and assembled in CANU assembler (version 1.8)[7] with default parameters. Due to the high heterozygosity of this genome, we further mapped the PacBio subreads against CANU initial contig assembly and identified primary contigs based on the read-depth strategy implemented in purge_haplotigs[8]. To further correct systematic errors of PacBio sequencing, 150 Gb (~50× coverage) of Illumina short reads were sequenced on the Illumina NovaSeq platform and mapped against nonredundant genome assembly using BWA-MEM (version 0.7.8)[9] with default parameters. Variants that were considered as sequencing errors were corrected using Pilon[10] with the following parameters: --mindepth 4 --threads 6 --tracks --changes --fix bases --verbose. Two high-quality Hi-C libraries were constructed[11]. Chimeric DNA fragments that represented sequences from proximal regions were detected based on Illumina paired-end sequencing model. We identified mis-assembled contigs that displayed abnormal long-rang contact patterns from paired-end reads alignments against the contig assembly using juicer tools[12] and the 3D-DNA pipeline[13]. The resulting contigs were then partitioned into nine groups, representing nine pseudo-chromosomes, using ALLHiC (version 0.9.8) with a diploid scaffolding model[14].

To annotate the protein-coding genes, we applied the same method as described previously in the sugarcane genome[15]. Briefly, we integrated evidences from orthologous proteins, transcriptomes and *ab initio* gene prediction using the MAKER pipeline[16]. In addition, we used RepeatMasker (http://www.repeatmasker.org/, version 4.0.5) and TEclassify[17] to annotate repetitive sequences.

Synteny analysis

Synteny analysis between the diploid *C. nankingense* genome and *C. morifolium* were performed using MCScan algorithm[18], which was implanted in JCVI package (https://github.com/tanghaibao/jcvi). Briefly, two files should be prepared for each species that was involved in the comparison, one FASTA file and one BED file. The FASTA file contains coding sequences for the species, while the BED file recorded gene information, including gene names and position located in chromosomes. These files were subjected to the following JCVI command line: python -m jcvi.compara.catalog ortholog.

Supplementary Note 3. Genome annotation of *C. morifolium*

Identification of protein-coding genes

To predict protein-coding genes in the *C. morifolium* genome, we used homology-based prediction, *de novo* prediction and transcriptome-based prediction. Homologue proteins from six plant genomes (*Arabidopsis thaliana, Daucus carota, Helianthus annuus, Lactuca sativa, Solanum lycopersicum* and *Solanum tuberosum*) were downloaded from Ensembl Plants (http://plants.ensembl.org/index.html) and NCBI (https://www.ncbi.nlm.nih.gov/). Protein sequences from these genomes were aligned to the *C. morifolium* genome assembly using TBLASTN[19], with an *E*-value cut-off of 1e-5. The BLAST hits were conjoined by Solar software[20]. GeneWise (https://www.ebi.ac.uk/Tools/psa/genewise, version 2.4.1)[21] was used to predict the exact gene structure of the corresponding genomic regions in each BLAST hit (Homo-set). For transcriptome-based prediction methods, RNA-seq data from three tissues (root, stem and leaf) were mapped onto the assembly using TopHat (http://ccb.jhu. edu/software/tophat/index.shtml, version 2.0.8) (--splice-mismatches 2 --max-intron-length 500000 --min-intron-length 50) and Cufflinks (http://cole-trapnell-lab.github.io/cufflinks/, version 2.1.1)[22, 23] (--max-intron-length 500000 --min-intron-length 50 --max-mle-iterations 5000). In addition, Trinity was used to assemble the RNA-seq data with the following parameters: "--min_glue 2 --min_kmer_cov 2", and the result was used to create several pseudo-unigenes. These pseudo-unigenes were also mapped onto the assembly, and gene models were predicted by PASA (http://pasapipeline.github.io/)[24]. This gene set was denoted PASA-T-set and was used to train *ab initio* gene prediction programs. Five *ab initio* gene prediction programs, namely, Augustus (http://augustus.gobics.de/, version 3.2.3), GENSCAN (http://genes.mit.edu/GENSCAN.html, version 1.0), GlimmerHMM (http://ccb.jhu.edu/software/glimmerhmm/, version 3.0.1), Geneid (http://genome.crg.es/software/geneid/, version 1.4), and SNAP (http://korflab.ucdavis.edu/software.html, version 2013-11-29), were used to predict coding regions in the repeat-masked genome[25, 26] with default parameters. Gene model evidence from Homo-set, Cufflinks-set, PASA-T-set and *ab initio* programs were combined by EVidenceModeler (EVM)

(http://evidencemodeler.sourceforge.net/, version 1.1.1)[27] to produce the nonredundant set of gene structures.

Non-coding RNA annotation

We annotated non-coding RNAs (ncRNAs) using several databases and software packages. Firstly, tRNA genes were identified by tRNAscan-SE software[28] with default parameters. Then, the ribosomal RNAs (rRNA) were predicted by aligning to the rRNA sequences using BLASTn at *E*-value of 1e-10. The miRNA and snRNA genes were predicted by INFERNAL software[29] against the Rfam database (release 9.1)[30].

Functional annotation of protein-coding genes

Functional annotation of protein-coding genes was achieved by using BLASTP (*E*-value: 1e-5) [31] against two integrated protein sequence databases: SwissProt (http://web.expasy.org/docs/swiss-prot_guideline.html) and NR (ftp://ftp.ncbi.nih.gov/blast/db/). Protein domains were annotated by searching against the InterPro ((http://www.ebi.ac.uk/interpro/, version 32.0) and Pfam (http://pfam.xfam.org/, version 27.0) databases, using InterProScan (version 4.8) and HMMER (http://www.hmmer.org/, version 3.1), respectively[32-35]. The Gene Ontology (GO, http://www.geneontology.org/page/go-database) terms for each gene were obtained from the corresponding InterPro or Pfam entry. The pathways in which the genes might be involved were assigned by BLAST against the Kyoto Encyclopedia of Genes and Genomes (KEGG) database (http://www.kegg.jp/kegg/kegg1.html), with an *E*-value cut-off of 1e-5. To compare the protein families among *C. morifolium,* six Asteraceae sequenced plants and *Arabidopsis thaliana* (Supplementary Figs. 10, 11 and 25), an HMM search was conducted using two Pfam domains PF01397 (N-terminal) and PF03936 (C-terminal) as seed sequence to identify the TPSs. The other two Pfam domains PF00319 (SRF-TF) and PF01486 (K-box) were used to search the MADS-box proteins. The target hits (*E*-value < 1e-5) with one or two of the domains were retrieved as TPS or MADS-box candidate genes. Meanwhile, manual curation and validation of these TPS or MADS-box candidates were performed using each candidate genes as queries to do BLASTp against the NCBI database and used for further analysis.

Identification of transposable elements

Transposable elements (TEs) in the *C. morifolium* genome were identified by combining *de novo*-based and homology-based approaches. For the *de novo*-based approach, we used RepeatModeler (http://www.repeatmasker.org/RepeatModeler.html, version 1.0.8)[36] with parameters of “-engine ncbi -pa 15”, and a *de novo* repeat family identification and modelling package, LTR_FINDER (http://tlife.fudan.edu.cn/ltr_finder/)[37] was used to build a *de novo* repeat library under default parameters (-C -w 2). For the homology-based approach, we used RepeatMasker (http://www.repeatmasker.org, version 3.3.0) with the parameters: “-a -nolow -no_is -norna” against

the Repbase TE library and RepeatProteinMask (http://www.repeatmasker.org/, version 4.0.5) with the parameters "-noLowSimple -pvalue 0.0001 -engine wublast" against the TE protein database[38]. To more accurately identity the LTR retrotransposons (LTR-RTs), we used LTRharvest[39] and LTR_FINDER (version 1.0.7) [37] to predict LTR-RTs with the following parameters: LTR length of 100 - 5,000 base pairs (bp), LTR interspace length of 1,000-20,000 bp. LTRdigest (version 1.5.8)[40] was used for structure annotation (e.g., PBS, PPT, protein) with optimal annotation. Candidate LTRs that were classified into *Gypsy* and *Copia* superfamilies were processed into activity analysis.

Estimation of the LTRs insertion time

For the intact LTR-RTs, we performed alignment of the sequences between the 5′ and 3′ LTRs using MUSCLE (version 3.8.31)[41], and nucleotide variations (λ) in 5′ and 3′ of intact LTR-RTs were calculated. Then the DNA substitution rates (K) were calculated by K = -0.75ln(1-4λ/3). Finally, the insert time of LTR-RTs was estimated using the formula $T = K/2r$, where r refers to a general substitution rate of 1.3×10^{-8} per site per year in Asteraceae family.

Supplementary Note 4. Genome evolution

Phylogenetic analysis

The protein sequences of *Artemisia annua*, *Aquilegia coerulea*, *Amborella trichopoda*, *Coffea canephora*, *Cynara cardunculus*, *Chrysanthemum seticuspe*, *Daucus carota*, *Helianthus annuus*, *Lactuca sativa*, *Oryza sativa*, *Solanum lycopersicum*, *Vitis vinifera* and *Zea mays* were downloaded from Ensembl Plants (http://plants.ensembl.org/index.html), and NCBI (https://www.ncbi.nlm.nih.gov/). Here, the constructed nonredundant consensus gene set that contained 94,552 genes for *C. morifolium* and the total 37,995 protein-coding genes of *Chrysanthemum nankingense* genome assembly in the present study (Supplementary Data 3) were respectively used for subsequent analysis.

All the genes of the 15 species were filtered as follows: (a) when multiple transcripts were present in one gene, only the longest transcript in the coding region was taken for further analysis, and (b) the genes encoding proteins with fewer than 30 amino acids were filtered out. We obtained the similarities between the protein sequences of all species through BLASTp with an E-value < 1e-5. The protein sequences of all 15 species were clustered into paralogues and orthologues using the program OrthoMCL (http://orthomcl.org/orthomcl/, version 0.5.1) with an inflation parameter equal to 1.5. After gene family clustering, we aligned 491 single-copy gene protein sequences by MUSCLE[41] and combined all the alignment results into a super-alignment matrix. Then, a phylogenetic tree of the 15 species was constructed using RAxML (http://sco.h-its.org/exelixis/web/software/raxml/index.html, version 8.0.19) with the maximum likelihood

method and 100 bootstrap replicates[42]. *A. trichopoda* was used as outgroups in the phylogenetic tree. Finally, the MCMCTree program (http://abacus.gene.ucl.ac.uk/software/paml.html, version 4.5) implemented in Phylogenetic Analysis by Maximum Likelihood (PAML) was applied to infer divergence time based on the phylogenetic tree[43]. The MCMCTree run parameters were a burn-in of 10,000, sample number of 100,000, and sample frequency of 2. The calibration time of divergence between *C.cardunculus* and *L.sativa* was 25.0-43.0 Million years ago (Mya), *C.nankingense* and *C.seticuspe* was 3 Mya, *A.annua* and *C.cardunculus* was 32.0-41.0 Mya, *S.lycopersicum* and *C.canephora* was 77.0-91.0 Mya, *V.vinifera* and *A.annua* was 110.0-124.0 Mya, *O.sativa* and *Z.mays* was 40.0-53.0 Mya, monocots and Dicotyledons was 148.0-173.0 Mya, and *A.trichopoda* and angiosperms was 168.0-194.0 Mya, according to the TimeTree database (http://www.timetree.org/).

Expansion and contraction of gene families

We determined the expansion and contraction of the gene families by comparing the cluster size differences between the ancestor and each species using the CAFÉ program (version 2.1)[44]. A random birth and death model was used to evaluate the changes in gene families along each lineage of the phylogenetic tree. A probabilistic graphical model (PGM) was introduced to calculate the probability of transitions in gene family size from parent to child nodes in the phylogeny. Using conditional likelihoods as the test statistics, we calculated the corresponding *P*-values in each lineage, and a *P*-value < 0.05 was used to identify families that had significantly expanded and contracted.

Genome synteny and whole-genome duplication

To identify syntenic blocks, the protein sequences from *C. morifolium, C. carduncul*us, *C. nankingense*, *C. seticuspe*, and *H. annuus* were searched against themselves using BLASTp (*E*-value < 1e-5). The results were subjected to MCScanX (-a, -e:1e-5, -u:1, -s:5) to determine syntenic blocks[14]. At least five genes were required to define synteny. Then, the synonymous substitutions per synonymous site (*Ks*) values were calculated via KaKs_Calculator (version 2.0)[45] for each gene pair in the aligned blocks. The distributions of all *Ks* values were plotted via the R software and ggplot2 package to infer whole-genome duplication or speciation events that occurred during the evolutionary history. The peak *Ks* values were converted to divergence time according to the formula $T = Ks/2\lambda$, where T refers to divergence time; λ refers to the synonymous substitution rate of 8.25×10^{-9} mutations per site per year for asterids. The dot plots between *C. carduncul*us and *C. morifolium* as well as the *C. nankingense* genome were generated with Quota synteny alignment software to visualize the palaeopolyploidy events.

Gene duplication analysis

Different modes of gene duplication as whole-genome duplicates (WGD), tandem duplicates (TD), proximal duplicates (less than 10 gene distance on the same chromosome: PD), transposed duplicates (transposed gene duplications: TRD), or dispersed duplicates (other duplicates than WGD, TD, PD

and TRD: DSD) were identified using DupGen_finder[46] with default parameters. Then, the *Ka* (number of substitutions per nonsynonymous site), *Ks* (number of substitutions per synonymous site), and *Ka*/*Ks* values were estimated for gene pairs generated by different modes of duplication based on the MYN model in KaKs_Calculator (version 2.0)[45].

Supplementary Note 5. Origin of cultivated chrysanthemum

Sample selection, genome resequencing and phylogenetic analysis

China is recognized as the country of origin of cultivated chrysanthemum and the lower-middle reaches of the Yangtze River areas including Chongqing, Hubei, Jiangsu, Anhui as well as Henan were the most likely centers of origin[47]. Several wild *Chrysanthemum* species are speculated to be involved in the origin of cultivated chrysanthemum[47]. Based on the reference genome, we resequenced 12 representative wild *Chrysanthemum* species including almost all Chinese diploid wild species as well as several *C. indicum* with multiple ploidies (diploid and tetraploid) that have been reported to be the potential ancestors of cultivated chrysanthemum, to further investigate the origin of chrysanthemum.

The high-quality PE reads of 12 wild *Chrysanthemum* species were mapped onto the *C. morifolium* reference genome using BWA (version 0.7.8)[9] with the command 'mem -t 4 -k 32 -M'. In order to reduce mismatch generated by PCR amplification before sequencing, duplicated reads were removed by SAMtools[48]. After alignment, we performed SNP calling on a population scale with SAMtools pipeline (-q 1 -C 50 -t SP -t DP -m 2 -F 0.002)[48]. Then, to exclude SNP calling errors caused by incorrect mapping, only 11,755 high-quality SNPs (coverage depth $\geq$ 20 & RMS mapping quality $\geq$20 & MAF $\geq$ 0.05 & miss $\leq$ 0.1) were kept for subsequent analysis. Using *H. annuus* as an outgroup, we constructed a maximum likelihood (ML) phylogenetic tree using IQ-TREE (version 1.6.12)[49], according to the best model determined by the Bayesian information criterion (BIC). The reliability of the ML tree was estimated using the ultrafast bootstrap approach (UFboot) with 1000 replicates. To further reveal the phylogenetic relationships within a homoeologous chromosome set, the chromosome-unique SNPs were isolated and employed for ML tree construction using the same approach (Supplementary Figure 17). An online tool Interactive tree of life (iTOL) v6 (https://itol.embl.de) was used to visualize the ML tree.

To preliminarily associate the SNP variation with phenotypic features, we counted the number of high-quality SNPs in 1 Mb non-overlapping sliding windows. The genes within the top 5% hot spots of SNP distribution regions were selected and used to perform GO enrichments analysis. The results showed that the 'auxin homeostasis' and 'regulation of flavonoid biosynthetic process' were the most enriched biological processes terms that might respectively involve floral development and colouration (Supplementary Data 13).

Statistical analysis of sequencing depth and IS calculation

In order to detect the introgressed sequences from wild *Chrysanthemum* species to cultivated chrysanthemum, we counted the unique and shared 100-kb non-overlapping windows with mapping depth larger than 4x among the 12 *Chrysanthemum* species. The identical score (IS) values were calculated based on SNP density with 100 kb windows. We calculated ISs to evaluate the similarities of the sequenced genomes to the 'Zhongshanzigui' reference genome according to Ai *et al.* [50].

$$\mathrm{IS} = \frac{\sum_{i=1}^{n} Si}{2(n-n')} \qquad (2)$$

where *Si* is the number of alleles identical to the 'Zhongshanzigui' reference allele at a certain SNP site *i*, *n* and *n'* refer the total number of SNPs and missing SNPs within a 100-kb window, respectively.

Gene retention analysis

Orthologues between *C. morifolium* and *C. nankingense* genomes assembled in this study, as well as between *C. morifolium* and *C. seticuspe*[2] were respectively determined by MCScanX using default settings (-a, -e:1e-5, -u:1, -s:5). A sliding window approach with window size of 100 syntenic genes and a step size of 10 genes by respectively using the *C. nankingense* and *C. seticuspe* genome as the reference was employed to calculate the proportion of the retained genes in *C. morifolium*.

Identification of chromosome-specific *K*-mers

To look for evidence that the *C. morifolium* genome could be partitioned into three distinct subgenomes based on distinctive histories, we scanned these pairs of chromosomes for 13-bp sequences (13-mers) that were found in many copies across genome, occurring at least 100 times across the whole genome, and for each homoeologous pair, were at least two-fold enriched in one member relative to either two pseudochromosomes. In addition, we used the Smudgeplot (https://github.com/KamilSJaron/smudgeplot) method[51] to visualize and estimate the ploidy and genome structure of *C. morifolium* by analyzing heterozygous *K*-mer pairs.

Meiotic and mitotic behaviors of the sequenced haploid and doubled haploid

We observed the meiosis behaviors of the sequenced haploid and colchicine induced doubled haploid that generated by Wang *et al.*[1]. The chromosomes of haploid and doubled haploid plants were obtained from anthers, which were obtained from 3 mm floral buds in sunny morning. Floral buds were fixed in 3:1 methanol : acetic acid solution overnight at 4°C. Then, the floral buds were washed in ddH_2O and stored at -20°C. The PMCs (pollen mother cells) dissected from tube florals on the slide was then squashed in 10 μL 45% acetic acid solution. Next, the meiotic slides conducted flame drying for examination.

For mitotic slides preparation, the plants were reproduced in a 3:1 mixture of garden soil and

vermiculite by approximately 5 cm stem cutting containing terminal bud. Then the rooting seedlings were planted in a greenhouse with 70% relative humidity at 25°C/20°C (day/night). The prepared plant root tips were squashed in 10 μL 45% acetic acid solution then flame drying for observing[52]. For statistical analysis, at least 30 cells of each plants were observed, and karyotypes were generally from a single cell.

Plant material and metaphase chromosome preparation for FISH analysis

To further investigate the genome structure of the sequenced diploid plant of *C. morifolium* cv. 'Zhongshanzigui', we conducted a FISH (fluorescence in situ hybridization) analysis. Healthy, uniform cuttings of each entry were planted in Styrofoam nursery trays with 72 caves containing a 2:2:1 mixture of perlite, vermiculite, and leaf mold. Rooted seedlings were transplanted into a small pot and grown in a greenhouse under a natural light at 22°C during the day and 15°C at night, with a relative humidity range of 70%. The pretreated the section of root-tips containing dividing cells was cut and digested in 20 μL 1% pectolyase Y-23 (Yakult, Japan, Tokyo cat. # MX7354) and 2% cellulase Onozuka R-10 solution (Yakult, Japan, Tokyo cat. # MX7352) for 1 h at 37°C. After the digestion treatment, the dividing sections of root tip were washed in 75% ethanol two times briefly. The root sections were carefully broken using a dissecting needles and collected by centrifugation (2,400 g for 30 sec). The precipitation was resuspended in 100% acetic acid solution. Finally, the cell suspension was dropped onto glass slides (10 μL per slide) in a wet box and dried slowly.

Oligo probe design and Oligo-FISH

The identification of repetitive sequences was conducted by RepeatExplore2 (https://repeatexplorer-elixir.cerit-sc.cz/galaxy) -TAREAN based on 1.2 million randomly selected Illumina sequence reads. The TAREAN analysis was run with default parameters according to the protocol in guidance of RepeatExplore2[53]. To ascertain the localization of 12 putative satellites in *C. morifolium,* single-strand oligonucleotides (25-40 nt) were designed from all satellite cluster results by Oligo 7 [54], synthetized and the 5' ends modified by TAMRA (6-carboxytetramethylrhodamine) or FAM (6-carboxyfluorescein) at General Biosystems (Chuzhou, Anhui Province, China). All the designed oligonucleotides probes were diluted to 0.55 ng/μL for use and the nucleotide sequences were given in Supplementary Data 8.

Firstly, we employed the examination of designed oligo probes by using Oligo-FISH and got the oligo probes with distinct signals. The Oligo-FISH was conducted as described in our previous study with minor modifications[55]. Briefly, the spreads were subjected to UV-crosslinked treatment (total energy, 120 mJ/cm2) after drying on slides. Then, two oligonucleotide cocktails CmOP-1(CL22, CL110 and CL151, modified by FAM at 5' ends) and CmOP-2(CL77, CL143, CL173 and CL263, modified by FAM at 5' ends) were mixed by equal amount of each oligo probes. At the center of the cell spreads, 10.0 μL hybridization solution per spread containing 1.0 μL CmOP-1 probe (0.55 ng/μL),

1.0 μL CmOP-2 probe (0.55 ng/μL), and 8.0 μL buffer (equal amount of 1 × TE and 2 × SSC) was dropped. After the application of a plastic coverslip, the slide preparation was denatured by being placed on a wet paper towel in an aluminum tray floating in boiling water (100℃) for 5 min in dark conditions. Next, the slides were incubated at 55℃ overnight in a humidity chamber containing 2 × SSC soaked paper toweling. The next day, slides were washed in 2 × SSC for 5 min at room temperature, and mounted with DAPI mounting medium (H-1200, Vector Laboratories, Burlingame, CA, USA) after drying while we waited to make observations.

Karyotyping analysis

Images were captured using a SPOT CCD camera (SPOT Cooled Color Digital, Olympus DP72, Tokyo, Japan). Then, multi-color component images were merged using Cellsens Dimension software (version 1.6). For karyotyping, 3-5 cells from each accession were observed, and karyotypes were generally obtained from a single cell. Otherwise, they were sampled from 1 to 4 cells because of overlapping chromosomes. The chromosome idiograms was draw using KaryoMeasure software (version 1.6.4)[56] based on the Oligo-FISH result.

Supplementary Note 6. Homoeolog expression bias analysis

To perform the homoeolog expression bias analysis, we re-analyzed the RNA-seq datasets of different organs from our previously published paper[3] using the assembled *C. morifilum* genome as reference. The samples including root, stem, leaf, shoot apexes, buds of different developmental stages and flower organs that were extracted from the one-month-old plants of 'Jinba', a popular commercial spray-cut chrysanthemum cultivar. Among them， the whole buds with phyllaries of four different stages when their diameter was either < 2 mm (Bud_X2), ~ 2 mm (Bud_2), ~ 4 mm (Bud_4) or ~ 8 mm (Bud_8) were used for RNA extraction. The flower organs samples were, respectively, petal (R_Pe) and pistil (R_pi) of ray florets as well as petal (D_Pe), pistil (D_pi) and stamen (D_st) of disc florets, which were extracted during the early bloom stage of inflorescence development.

The Raw RNA reads downloaded under NCBI BioProject ID of PRJNA548460 were trimmed and mapped onto the draft reference genomes by TopHat[22] with the following parameters: --max-intron-length 500,000, --read-gap-length 10, --read-edit-dist 15, --max-insertion-length 5 and --max-deletion-length 5. The detailed mapping information of the samples could be found in Supplementary Table 8. To accurately quantify homoeologous gene expression, only the reads that uniquely mapped were kept for further analysis. The expression level (fragments per kilobase of transcript per million mapped reads, FPKM) of each protein-coding gene was calculated by using HTSeq[57] with default parameters. This average FPKM values across all the tissues were used to the subsequent expression bias analysis.

According to Ramírez-González *et al.* [58], we only focused exclusively on the 11,438 expressed gene triads (34,314 genes) that the sum FPKM of the three genes within a triad > 0.5, with the aim to include the triads in which, for example, only a single homoeolog was expressed. To standardize the relative expression of each homoeolog across the triad, we normalized the absolute FPKM for each gene within the triad as follows:

$$expression_{ChrX} = FPKM_{ChrX} / (FPKM_{ChrX} + FPKM_{ChrY} + FPKM_{ChrZ}) \quad \textbf{(3)}$$

$$expression_{ChrY} = FPKM_{ChrY} / (FPKM_{ChrX} + FPKM_{ChrY} + FPKM_{ChrZ}) \quad \textbf{(4)}$$

$$expression_{ChrZ} = FPKM_{ChrZ} / (FPKM_{ChrX} + FPKM_{ChrY} + FPKM_{ChrZ}) \quad \textbf{(5)}$$

Where Chr*X*, Chr*Y* and Chr*Z* represent the three homoeologous chromosomes within in a given triad. Subsequently, we defined seven homoeolog expression bias categories and determined a triad's position in the ternary plot according to the relative contributions of each homoeolog: a balanced category, with similar relative abundance of transcripts from the three homoeologs, and six homoeolog dominant or homoeolog suppressed categories, classified on the basis of the higher or lower abundance of transcripts from a single homoeolog with respect to those from the other two (Supplementary Figure 24). The values of the relative contributions of each triad within a homoeologous group were visualized using the R package ggtern[59]. Go enrichment analysis of the balanced genes for each triad was respectively performed in GOseq software[60]. The significance of enrichment was valued against the background syntenic genes using a Fisher's exact test.

To further investigate if the gene orientation change has any implication for biological processes, we firstly analyzed synteny in each of the nine homoeologous chromosome groups and observed a greater difference of synteny in inter-group (25.3% to 40.8%) than in intra-group (Supplementary Table 15). About the orientation of the synteny genes in gene locus, we found 79.7% to 94.6% of them to be the same (Supplementary Table 15). Further, we performed a correlation analysis between the synteny gene order change and differential gene expression (Supplementary Table 16).

Supplementary Note 7. Mining of flower shape related candidate genes

BSA-seq analysis for flower shape

DNA pools construction

An F_1 population was generated from a cross between maternal flat petal type 'Hongxiao' (HX) and paternal tubular petal type 'Q5-12' (n = 179) using artificial hybridization in 2019 (Figure 4a). All materials were stored at the Chrysanthemum Germplasm Resource Preserving Centre, Nanjing, Jiangsu Province, China. We investigated the ray floret shape related traits for F_1 plants and two parents in the fall of 2020. The ratio of the corolla tube length and ray floret length was used to quantitatively describe the corolla tube merged degree (CTMD). For BSA-seq analysis, two

extreme DNA pools, flat-bulk (BF) and tubular-bulk (BT), were constructed, respectively, by mixing an equal amount of DNA from 20 individuals with flat petal types (CTMD = 0.07-0.13) and 20 individuals with tubular petal types (CTMD = 0.70-0.93) (Supplementary Figure 27).

Resequencing and variants calling

Four DNA libraries including two parents and two progeny pools were sequenced on DNBseq-T7 platform to obtain 150 bp PE reads at an average of 30× coverage. A total of 1,879,376,876, 1,938,040,136, 1,873,630,478 and 1,801,692,788 short clean reads were produced for HX, Q5-12, BF and BT, respectively. Then, these short reads were aligned to the chrysanthemum reference genome assembled in this study using mem module of BWA software[9]. The mapping rates were between 98.19-98.84% of the reference genome among samples, with 1× coverage more than 94.26%. The Unified Genotyper function in GATK software[61] was used for SNP/InDel variants calling. The raw variants were further filtered using GATK's Variant Filtration with appropriate parameter settings (-Window 4, -filter 'QD < 4.0 || FS > 60.0 || MQ < 40.0', -G_filter 'GQ < 20').

BSA-seq analysis

Additionally, SNPs and InDels that (1) exhibited uniparental (nn × np and lm × ll) as well as bi-parental (hk × hk) inheritance; (2) have no missing genotypes; (3) with read depth > 5x in per parent, while between 10x and 500x for each pool were retained for BSA-seq analysis. To reduce ambiguity introduced by sequencing error, markers with SNP-index < 0.3 or > 0.7 in both bulks were also discarded. Thus, 6,816,214 SNPs and 606,557 InDels were used to calculated SNP-index[62, 63] and Euclidean distance (ED)[64]. Regions of the genome representing the top 1% of absolute ΔSNP index and ED^2 values were considered to be strongly associated with the corolla tube merged degree, using a sliding window of 500 kb with 50 kb step.

WGCNA analysis for flower shape

Three flat petal type cultivars ('Nannongxixia', 'Nannongqingyu', 'Qinhuaijinhui'), three tubular petal type cultivars ('Nanongxuesong', 'Anastasia Brown', 'Xuesongyue'), and three spoon petal type cultivars ('Nannonglifengche', 'Nannongziyu', 'Nannongziyunjian') were selected to conduct RNA-seq analysis (Supplementary Figure 25). Among these, 'Nannongxixia', 'Nannongqingyu', 'Nanongxuesong', 'Nannonglifengche' and 'Nannongziyu' are anemone-type chrysanthemums, featuring elongated and pigmented disk florets. The ray florets and disc florets were dissected at the early bloom stage for RNA extraction and transcriptome sequencing. Notably, the disc florets of 'Anastasia Brown' have become vestigial.

Differential gene expression analysis between each two groups of the three petal types was performed by DESeq[65]. Genes with a corrected *p*-values less than 0.05 and |log2 Fold Change| ≥ 1 were considered to be differentially expressed genes between different groups. Co-expression

networks were constructed using WGCNA (version 3.2.5)[66] package in R. After filtering low-abundance (FPKM ≤ 1) genes of samples, 74,074 ray-specific were performed for WGCNA analysis, respectively, using a dynamic tree cut algorithm with a minimum module size of 50 genes and a merging threshold of 0.25. The CTMD values were used as phenotypic data to identify petal type related modules. Co-expression networks were visualized in Cytoscape software (version 3.6.1)[67].

Supplementary Note 8. The biosynthesis pathways of anthocyanin and flavonol in chrysanthemum

Plant material

Flower colour is a major objective of ornamental plant breeding. To investigate the biosynthesis pathway of flower pigments in chrysanthemum, three Santini series chrysanthemum cultivars with similar genetic background but different flower colours, i.e., 'Mini Pink' (Mini P), 'Mini Yellow' (Mini Y) and 'Mini White' (Mini W), were used (Supplementary Figure 30a). The outermost two ray floral whorls for each cultivar were sampled from the inflorescences (~12 mm in diameter) at early bloom stage and immediately frozen by liquid nitrogen with three biological replicates. All samples were stored at -80°C for use.

Relative pigment content measurement

The pigment contents of three cultivars were analyzed by a spectrophotometry method. Firstly, the fresh samples were weighed and immediately ground into powder. Three biological replicates were prepared for each cultivar. After transferred the petal powder into 5 mL tubes, 3 mL extractant contained acetone and petroleum ether (1:4, *v*/*v*) was added for carotenoids extraction. The carotenoids were released under dark soaking at 4°C for 48 h. The supernatant was taken after centrifuging at 1,500 g for 10 min. Taking the extractant as the blank control, the absorbance was calculated at 450 nm. Relative carotenoids content was quantified by formula:

$$c\ (\mathrm{mg/g}) = (A_{450} \times N \times V)/(E1\%1\mathrm{cm} \times d \times m) \qquad \mathbf{(6)}$$

where A_{450} represents the absorbance value at 450 nm, *N* is dilution multiple, *V* is the volume of extractant, E1%1 cm is carotenoid absorbance coefficient (use mean value of 2500), *d* represent cuvette inner diameter (usually 1 cm), *m* is the sample weight. As a result, a significant higher relative carotenoids content in 'Mini Yellow' than that in 'Mini Pink' and 'Mini White' was observed (Supplementary Figure 30c).

Anthocyanin content measurements using the similar protocol. The anthocyanins were released by the extractant contained methanol : water : formic acid : TFA (70:27:2:1, *v*/*v*/*v*/*v*) under dark soaking at 4°C for 24 h. The supernatant was taken after centrifuging at 1,500 g for 10 min. Taking the

extractant as the blank control, the absorbance was calculated at 530 nm. Relative total anthocyanins content was quantified by formula:

$$c\ (\mathrm{OD/g}) = A_{530} \times N \times V/m \qquad (7)$$

where A_{530} represents the absorbance value at 530 nm, N is dilution multiple, V is the volume of extractant, m is the sample weight. As expected, the total anthocyanins content of 'Mini Pink' was significantly higher than 'Mini Yellow' and 'Mini White' (Supplementary Figure 30b).

Gene expression analysis

The total RNA was extracted using plant RNA Isolation Kit (Huayueyang, Beijing, China). The RNA-seq was performed on Illumina HiSeqTM 4000 instrument by Gene Denovo Biotechnology Co. (Guangzhou, Guangdong Province, China). Based on the chromosome-scale genome sequence of *C. morifolium*, the bioinformatic analysis was conducted as described in Supplementary Note 6. Since there were no *CCD4a* genes in 'Zhongshanzigui' genome, *de novo* transcriptome assembly was performed by Trinity[68] using clean reads that filtered the raw reads containing adapters, unknown nucleotides (> 10%), and low-quality reads with the percentage of Q-value ($\leq$ 20) base higher than 50%. After assembly, five functional databases (NR, GO, COG/KOG, KEGG and SwissProt) were used to annotating unigenes, and unigene expression was quantified in FPKM values.

A transcriptional view of the biosynthesis pathways of flavonoids

The high-quality *C. morifolium* genome assembly allowed reconstruction of the metabolic pathway for flower coloration by capturing the implicated enzymatic genes in this process. It is reported that flavonoids are synthesized by a branched pathway that yields both colored anthocyanin pigments and colorless flavonols[69]. Here, we unveiled 25 genes encoding 9 enzymes functioning in anthocyanin and flavonol biosynthesis (Supplementary Figure 30d).

Our results showed that the expression levels of anthocyanin biosynthesis genes in 'Mini Pink' were generally higher than that of 'Mini Yellow' and 'Mini White'. The expression level of *CmFLS* was distinct in 'Mini Yellow', suggesting that the highly expressed of *CmFLS* in 'Mini Yellow' competed with the same substrate of the flavonol pathway and anthocyanin pathway, thus resulting in more colorless flavonols, which contributed to its lack of pink. Since there were no *CCD4a* genes annotated in the 'Zhongshanzigui' reference genome, we further made a *de novo* transcriptome assembly and found that the expression of *CmCCD4a* in 'Mini Yellow' was significantly lower than that of 'Mini Pink' and 'Mini White', which was consistent with the carotenoid content in the three cultivars (Supplementary Figure 30c, e). Collectively, our results indicate that the flower colour diversity is a combined consequence of anthocyanin and carotenoid metabolic pathways in chrysanthemum.

Supplementary references

1. Wang, H. *et al*. Characterization of *in vitro* haploid and doubled haploid *Chrysanthemum morifolium* plants via unfertilized ovule culture for phenotypical traits and DNA methylation pattern. *Front. Plant Sci.* **5**, 738 (2014).
2. Nakano, M. *et al*. A chromosome-level genome sequence of *Chrysanthemum seticuspe*, a model species for hexaploid cultivated chrysanthemum. *Commun. Biol.* **4**, 1-11 (2021).
3. Ding, L. *et al*. The core regulatory networks and hub genes regulating flower development in *Chrysanthemum morifolium*. *Plant Mol. Biol.* **103**, 669-688 (2020).
4. Yaffe, E. & Tanay, A. Probabilistic modeling of Hi-C contact maps eliminates systematic biases to characterize global chromosomal architecture. *Nat. Genet.* **43**, 1059-1065 (2011).
5. Liu, B. *et al*. Estimation of genomic characteristics by analyzing k-mer frequency in *de novo* genome projects. Preprint at https://arxiv.org/abs/1308 (2012).
6. Zhang, Q. *et al*. The genome of *Prunus mume*. *Nat. Commun.* **3**, 1318 (2012).
7. Koren, S. *et al*. Canu: scalable and accurate long-read assembly via adaptive k-mer weighting and repeat separation. *Genome Res.* **27**, 722-736 (2017).
8. Roach, M.J., Schmidt, S.A. & Borneman, A.R. Purge Haplotigs: allelic contig reassignment for third-gen diploid genome assemblies. *BMC Bioinformatics* **19**, 1-10 (2018).
9. Li, H. Toward better understanding of artifacts in variant calling from high-coverage samples. *Bioinformatics* **30**, 2843-2851 (2014).
10. Walker, B.J. *et al*. Pilon: an integrated tool for comprehensive microbial variant detection and genome assembly improvement. *PLoS One* **9**, e112963 (2014).
11. Xie, T. *et al. De novo* plant genome assembly based on chromatin interactions: a case study of *Arabidopsis thaliana*. *Mol. Plant* **8**, 489-492 (2015).
12. Durand, N.C. *et al.* Juicer provides a one-click system for analyzing loop-resolution Hi-C experiments. *Cell Syst.* **3**, 95-98 (2016).
13. Dudchenko, O. *et al*. *De novo* assembly of the *Aedes aegypti* genome using Hi-C yields chromosome-length scaffolds. *Science* **356**, 92-95 (2017).
14. Zhang, X., Zhang, S., Zhao, Q., Ming, R. & Tang, H. Assembly of allele-aware, chromosomal-scale autopolyploid genomes based on Hi-C data. *Nat. Plants* **5**, 833-845 (2019).
15. Zhang, J. *et al*. Allele-defined genome of the autopolyploid sugarcane *Saccharum spontaneum* L. *Nat. Genet.* **50**, 1565-1573 (2018).
16. Cantarel, B.L. *et al*. MAKER: an easy-to-use annotation pipeline designed for emerging model organism genomes. *Genome Res.* **18**, 188-196 (2008).
17. Abrusán, G., Grundmann, N., DeMester, L. & Makalowski, W. TEclass-a tool for automated classification of unknown eukaryotic transposable elements. *Bioinformatics* **25**, 1329-1330 (2009).
18. Tang, H. *et al*. Synteny and collinearity in plant genomes. *Science* **320**, 486-488 (2008).
19. Altschul, S.F., Gish, W., Miller, W., Myers, E.W. & Lipman, D.J. Basic local alignment search tool. *J. Mol. Biol.* **215**, 403-410 (1990).

20. Yu, X.-J., Zheng, H.-K., Wang, J., Wang, W. & Su, B. Detecting lineage-specific adaptive evolution of brain-expressed genes in human using rhesus macaque as outgroup. *Genomics* **88**, 745-751 (2006).
21. Cook, C.E., Bergman, M.T., Cochrane, G., Apweiler, R. & Birney, E. The European Bioinformatics Institute in 2017: data coordination and integration. *Nucleic Acids Res.* **46**, D21-D29 (2018).
22. Kim, D. *et al*. TopHat2: accurate alignment of transcriptomes in the presence of insertions, deletions and gene fusions. *Genome Biol.* **14**, 1-13 (2013).
23. Ghosh, S. & Chan, C. K. Analysis of RNA-seq data using TopHat and Cufflinks. *Methods Mol. Biol.* **1374**, 339-361 (2016).
24. Haas, B.J. *et al*. Improving the *Arabidopsis* genome annotation using maximal transcript alignment assemblies. *Nat. Protoc.* **31**, 5654-5666 (2003).
25. Keller, O., Kollmar, M., Stanke, M. & Waack, S. A novel hybrid gene prediction method employing protein multiple sequence alignments. *Bioinformatics* **27**, 757-763 (2011).
26. Blanco, E., Parra, G. & Guigó, R. Using geneid to identify genes. *Curr. Protoc. Bioinformatics* **18**, 1-4 (2007).
27. Haas, B.J. *et al*. Automated eukaryotic gene structure annotation using EVidenceModeler and the Program to Assemble Spliced Alignments. *Genome Biol.* **9**, 1-22 (2008).
28. Lowe, T.M. & Eddy, S.R. tRNAscan-SE: a program for improved detection of transfer RNA genes in genomic sequence. *Nucleic Acids Res.* **25**, 955-964 (1997).
29. Nawrocki, E.P., Kolbe, D.L. & Eddy, S.R. Infernal 1.0: inference of RNA alignments. *Bioinformatics* **25**, 1335-1337 (2009).
30. Griffiths-Jones, S. *et al*. Rfam: annotating non-coding RNAs in complete genomes. *Nucleic Acids Res.* **33**, D121-D124 (2005).
31. Altschul, S.F. *et al*. Gapped BLAST and PSI-BLAST: a new generation of protein database search programs. *Nucleic Acids Res.* **25**, 3389-3402 (1997).
32. Finn, R.D. *et al*. InterPro in 2017-beyond protein family and domain annotations. *Nucleic Acids Res.* **45**, D190-D199 (2017).
33. Finn, R.D. *et al.* The Pfam protein families database. *Nucleic Acids Res.* **38**, D211-D222 (2010).
34. Zdobnov, E.M. & Apweiler, R. InterProScan–an integration platform for the signature-recognition methods in InterPro. *Bioinformatics* **17**, 847-848 (2001).
35. Finn, R.D. *et al*. HMMER web server: 2015 update. *Nucleic Acids Res.* **43**, W30-W38 (2015).
36. Smit, A.F. & Hubley, R. RepeatModeler Open-1.0 (Institute for Systems Biology, 2008).
37. Xu, Z. & Wang, H. LTR_FINDER: an efficient tool for the prediction of full-length LTR retrotransposons. *Nucleic Acids Res.* **35**, W265-W268 (2007).
38. Chen, N. Using Repeat Masker to identify repetitive elements in genomic sequences. *Curr. Protoc. Bioinformatics* **5**, 4-10 (2004).
39. Ellinghaus, D., Kurtz, S. & Willhoeft, U. *LTRharvest*, an efficient and flexible software for *de novo* detection of LTR retrotransposons. *BMC Bioinformatics* **9**, 1-14 (2008).
40. Steinbiss, S., Willhoeft, U., Gremme, G. & Kurtz, S. Fine-grained annotation and classification of *de novo* predicted LTR retrotransposons. *Nucleic Acids Res.* **37**, 7002-7013 (2009).
41. Edgar, R.C. MUSCLE: multiple sequence alignment with high accuracy and high throughput.

Nucleic Acids Res. **32**, 1792-1797 (2004).
42. Stamatakis, A. RAxML version 8: a tool for phylogenetic analysis and post-analysis of large phylogenies. *Bioinformatics* **30**, 1312-1313 (2014).
43. Yang, Z. PAML 4: phylogenetic analysis by maximum likelihood. *Mol. Biol. Evol.* **24**, 1586-1591 (2007).
44. Han, M.V., Thomas, G.W., Lugo-Martinez, J. & Hahn, M.W. Estimating gene gain and loss rates in the presence of error in genome assembly and annotation using CAFE 3. *Mol. Biol. Evol.* **30**, 1987-1997 (2013).
45. Wang, D. *et al*. KaKs_Calculator 2.0: a toolkit incorporating gamma-series methods and sliding window strategies. *Genom. Proteom. Bioinf.* **8**, 77-80 (2010).
46. Qiao, X. *et al*. Gene duplication and evolution in recurring polyploidization–diploidization cycles in plants. *Genome Biol.* **20**, 1-23 (2019).
47. Chen, J., Wang, C., Zhao, H. & Zhou, J. The Origin of Garden Chrysanthemum (Anhui Science & Technology Publishing House, Hefei, 2012).
48. Li, H. *et al*. The sequence alignment/map format and SAMtools. *Bioinformatics* **25**, 2078-2079 (2009).
49. Nguyen, L. T., Schmidt, H. A., Von Haeseler, A. & Minh, B. Q. IQ-TREE: a fast and effective stochastic algorithm for estimating maximum-likelihood phylogenies. *Mol. Biol. Evol.* **32**, 268-274 (2015).
50. Ai, H. *et al*. Adaptation and possible ancient interspecies introgression in pigs identified by whole-genome sequencing. *Nat. Genet.* **47**, 217-225 (2015).
51. Ranallo-Benavidez, T.R., Jaron, K.S. & Schatz, M.C. GenomeScope 2.0 and Smudgeplot for reference-free profiling of polyploid genomes. *Nat. Commun.* **11**, 1-10 (2020).
52. He, J. *et al*. Identification of 5S and 45S rDNA sites in *Chrysanthemum* species by using oligonucleotide fluorescence in situ hybridization (Oligo-FISH). *Mol. Biol. Rep.* **48**, 21-31 (2021).
53. Novák, P., Neumann, P. & Macas, J. Global analysis of repetitive DNA from unassembled sequence reads using RepeatExplorer2. *Nat. Protoc.* **15**, 3745-3776 (2020).
54. Rychlik, W. OLIGO 7 primer analysis software. *Methods. Mol. Biol.* **402**, 35-60 (2007).
55. He, J. *et al*. Uneven levels of 5S and 45S rDNA site number and loci variations across wild *Chrysanthemum* accessions. *Genes* **13**, 894 (2022).
56. Mahmoudi, S. & Mirzaghaderi, G. Tools for drawing informative idiograms. Preprint at https://doi.org/10.1101/2021.09.29.459870 (2021).
57. Ramírez-González, R. *et al.* The transcriptional landscape of polyploid wheat. *Science* **361**, eaar6089 (2018).
58. Anders, S., Pyl, P.T. & Huber, W. HTSeq-a Python framework to work with high-throughput sequencing data. *Bioinformatic* **31**, 166-169 (2015).
59. Hamilton, N. ggtern: An extension to 'ggplot2', for the creation of ternary diagrams. Preprint at https://CRAN.R-project.org/package=ggtern (2016).
60. Young, M. D., Wakefield, M. J., Smyth, G. K. & Oshlack, A. Gene ontology analysis for RNA-seq: accounting for selection bias. *Genome Biol.* **11**, R14 (2010).
61. McKenna, A. *et al*. The genome analysis toolkit: a MapReduce framework for analyzing next-generation DNA sequencing data. *Genome Res.* **20**, 1297-1303 (2010).
62. Takagi, H. *et al*. QTL-seq: rapid mapping of quantitative trait loci in rice by whole genome

resequencing of DNA from two bulked populations. *Plant J.* **74**, 174-183 (2013).
63. Xue, H. *et al*. Interval mapping for red/green skin color in Asian pears using a modified QTL-seq method. *Hortic. Res.* **4**, 17053 (2017).
64. Hill, J.T. *et al.* MMAPPR: mutation mapping analysis pipeline for pooled RNA-seq. *Genome Res.* **23**, 687-697 (2013).
65. Anders, S. & Huber, W. Differential expression analysis for sequence count data. *Genome Biol.* **11**, R106 (2010).
66. Langfelder, P. & Horvath, S. WGCNA: an R package for weighted correlation network analysis. *BMC Bioinformatics* **9**, 1-13 (2008).
67. Shannon, P. *et al*. Cytoscape: a software environment for integrated models of biomolecular interaction networks. *Genome Res.* **13**, 2498-2504 (2003).
68. Haas, B.J. *et al*. *De novo* transcript sequence reconstruction from RNA-seq using the Trinity platform for reference generation and analysis. *Nat. Protoc.* **8**, 1494-1512 (2013).
69. Chen, H. *et al.* Allele-aware chromosome-level genome assembly and efficient transgene-free genome editing for the autotetraploid cultivated alfalfa. *Nat. Commun.* **11**, 1-11 (2020).
