# Supplementary material for: Analyses of a chromosome-scale genome assembly reveal the origin and evolution of cultivated chrysanthemum
Source: Nat Commun. 2023 Apr 11;14:2021. doi: 10.1038/s41467-023-37730-3 (PMC10085997; doi:10.1038/s41467-023-37730-3)
Supplement: Supplementary file 3 — Description of Additional Supplementary Files [file 41467_2023_37730_MOESM3_ESM.pdf]

Description of Additional Supplementary Files

File Name: Supplementary Data 1
Description: Comparisons of genome assemblies and annotations among sequenced Asteraceae species.

File Name: Supplementary Data 2
Description: General statistics of predicted protein-coding genes.

File Name: Supplementary Data 3
Description: The detailed genome information of 15 species used for comparative genomic analysis in this study.

File Name: Supplementary Data 4
Description: Enriched GO terms and KEGG pathways for gene families expanded in C. morifolium.

File Name: Supplementary Data 5
Description: Summary of the mapping ratio of 12 Chrysanthemum wild species.

File Name: Supplementary Data 6
Description: Genomic similarity of cultivated chrysanthemum and 12 Chrysanthemum wild species by identify score analysis.

File Name: Supplementary Data 7
Description: Gene retention ratios between C. nankingense and C. seticuspe in the C. morifolium genome.

File Name: Supplementary Data 8
Description: Oligonucleotide probes used for FISH analysis in this study

File Name: Supplementary Data 9
Description: Enriched GO terms for balanced genes in C. morifolium genome.

File Name: Supplementary Data 10
Description: List of 105 MADS-box genes identified in C. morifolium.

File Name: Supplementary Data 11
Description: Consensus QTLs for petal type via BSA-seq analysis.

File Name: Supplementary Data 12
Description: The identified auxin related genes via BSA-seq and WGCNA analyses.

File Name: Supplementary Data 13
Description: Enriched GO terms for genes within hot spots SNP distribution regions in C. morifolium genome.
