# Supplementary material for: Analyses of a chromosome-scale genome assembly reveal the origin and evolution of cultivated chrysanthemum
Source: Nat Commun. 2023 Apr 11;14:2021. doi: 10.1038/s41467-023-37730-3 (PMC10085997; doi:10.1038/s41467-023-37730-3)
Supplement: Supplementary file 5 — Reporting Summary [file 41467_2023_37730_MOESM5_ESM.pdf]

nature portfolio

Corresponding author(s): Jiafu Jiang, Sumei Chen, Fadi Chen

Last updated by author(s): Mar 14, 2023

Reporting Summary

Nature Portfolio wishes to improve the reproducibility of the work that we publish. This form provides structure for consistency and transparency in reporting. For further information on Nature Portfolio policies, see our Editorial Policies and the Editorial Policy Checklist.

Statistics

For all statistical analyses, confirm that the following items are present in the figure legend, table legend, main text, or Methods section.

n/a	Confirmed	
☐	☒	The exact sample size (*n*) for each experimental group/condition, given as a discrete number and unit of measurement
☐	☒	A statement on whether measurements were taken from distinct samples or whether the same sample was measured repeatedly
☐	☒	The statistical test(s) used AND whether they are one- or two-sided<br>*Only common tests should be described solely by name; describe more complex techniques in the Methods section.*
☒	☐	A description of all covariates tested
☐	☒	A description of any assumptions or corrections, such as tests of normality and adjustment for multiple comparisons
☐	☒	A full description of the statistical parameters including central tendency (e.g. means) or other basic estimates (e.g. regression coefficient) AND variation (e.g. standard deviation) or associated estimates of uncertainty (e.g. confidence intervals)
☐	☒	For null hypothesis testing, the test statistic (e.g. *F*, *t*, *r*) with confidence intervals, effect sizes, degrees of freedom and *P* value noted<br>*Give P values as exact values whenever suitable.*
☒	☐	For Bayesian analysis, information on the choice of priors and Markov chain Monte Carlo settings
☒	☐	For hierarchical and complex designs, identification of the appropriate level for tests and full reporting of outcomes
☐	☒	Estimates of effect sizes (e.g. Cohen's *d*, Pearson's *r*), indicating how they were calculated

Our web collection on statistics for biologists contains articles on many of the points above.

Software and code

Policy information about availability of computer code

Data collection	No software was used for data collection.
Data analysis	We used freely available bioinformatics software, including: FALCON (version 1.0), CANU (version 1.8), Quiver (version 2.0.0), ALLHiC (version 0.9.8), BWA (version 0.7.8), FragScaff (version 140324.1), Pilon (version 1.18), SAMtools (version 0.1.19), BLAT (version 35), GeneWise (version 2.4.1), CEGMA (version 2.5), BUSCO (version 4.1.2), tRNAscan-SE (version 2), INFERNAL (1.1rc4), RepeatModeler (version 1.0.8), LTRharvest (version 1.5.8), LTRdigest (version 1.5.8), LTR_FINDER (version 1.0.7), RepeatMasker (version 4.0.5), RepeatProteinMask (version 4.0.5), Blast (version 2.2.26), Solar (version 0.9.6), TopHat (version 2.0.8), Cufflinks (version 2.1.1), Trinity (version 2.1.1), Augustus (version 3.2.3), GENSCAN (version 1.0), GlimmerHMM (version 3.0.1), Geneid (version 1.4), SNAP (version 2013-11-29), EvidenceModeler (version 1.1.1), InterPro (version 32.0), Pfam (version 27.0), InterProScan (version 4.8), HMMER (version 3.1), OrthoMCL (version 0.5.1), MUSCLE (version 3.8.31), RAxML (version 8.0.19), MCMCtree (version 4.5), CAFÉ (version 2.1), MCScanX (version 2015), DupGen_finder (version 1.0), KaKs_Calculator (version 2.0), Cytoscape (version 3.6.1), IQ-TREE (version 1.6.12), Smudgeplot (version 0.2.4), RepeatExplore2 (version 2), Cellsens Dimension software (version 1.6), KaryoMeasure (version 1.6.4), GATK (version 3.8.0), WGCNA package (version 3.2.5) .

For manuscripts utilizing custom algorithms or software that are central to the research but not yet described in published literature, software must be made available to editors and reviewers. We strongly encourage code deposition in a community repository (e.g. GitHub). See the Nature Portfolio guidelines for submitting code & software for further information.

Data

Policy information about availability of data

All manuscripts must include a data availability statement. This statement should provide the following information, where applicable:

- Accession codes, unique identifiers, or web links for publicly available datasets
- A description of any restrictions on data availability
- For clinical datasets or third party data, please ensure that the statement adheres to our policy

The raw sequencing data generated in this study have been deposited in the NCBI under accession code PRJNA796762 [https://dataview.ncbi.nlm.nih.gov/object/PRJNA796762] and PRJNA895586 [https://dataview.ncbi.nlm.nih.gov/object/PRJNA895586]. The chloroplast and mitochondrial genome were also available at GenBank under the accession number OP104251 and OP104742, respectively. The assembled genome sequences and annotations are available at figshare (https://doi.org/10.6084/m9.figshare.21655364.v2), The query sequences are available at figshare (https://doi.org/10.6084/m9.figshare.21610305). Source data are provided with this paper.

Human research participants

Policy information about studies involving human research participants and Sex and Gender in Research.

Reporting on sex and gender	N/A
Population characteristics	N/A
Recruitment	N/A
Ethics oversight	N/A

Note that full information on the approval of the study protocol must also be provided in the manuscript.

Field-specific reporting

Please select the one below that is the best fit for your research. If you are not sure, read the appropriate sections before making your selection.

☒ Life sciences ☐ Behavioural & social sciences ☐ Ecological, evolutionary & environmental sciences

For a reference copy of the document with all sections, see nature.com/documents/nr-reporting-summary-flat.pdf

Life sciences study design

All studies must disclose on these points even when the disclosure is negative.

Sample size	One plant of Chrysanthemum morifolium was selected for genome sequencing and the same plant was used for RNA-seq of different organs for genome annotation. Differential gene expression analysis was performed in three individual plants as independent biological replicates.
Data exclusions	No data were excluded from the analyses.
Replication	Three biological replicates were used for RNA-seq and qPCR of gene expression studies. All attempts at replication were successful. One plant of each species was used for DNA sequencing, and all attempts were successful.
Randomization	Biological replicates were used for RNA-seq and qPCR of gene expression studies were randomly selected.
Blinding	Not applicable. The author did not do artificial group allocation in collecting samples, conducting the experiments and performing data analyses.

Reporting for specific materials, systems and methods

We require information from authors about some types of materials, experimental systems and methods used in many studies. Here, indicate whether each material, system or method listed is relevant to your study. If you are not sure if a list item applies to your research, read the appropriate section before selecting a response.

Materials & experimental systems

n/a	Involved in the study
☒	☐ Antibodies
☒	☐ Eukaryotic cell lines
☒	☐ Palaeontology and archaeology
☒	☐ Animals and other organisms
☒	☐ Clinical data
☒	☐ Dual use research of concern

Methods

n/a	Involved in the study
☒	☐ ChIP-seq
☒	☐ Flow cytometry
☒	☐ MRI-based neuroimaging
